# Biological Parts for Engineering Abiotic Stress Tolerance in Plants

**DOI:** 10.34133/2022/9819314

**Published:** 2022-01-21

**Authors:** Neeta Lohani, Mohan B. Singh, Prem L. Bhalla

**Affiliations:** Plant Molecular Biology and Biotechnology Laboratory, Faculty of Veterinary and Agricultural Sciences, The University of Melbourne, Parkville, VIC 3010, Australia

## Abstract

It is vital to ramp up crop production dramatically by 2050 due to the increasing global population and demand for food. However, with the climate change projections showing that droughts and heatwaves becoming common in much of the globe, there is a severe threat of a sharp decline in crop yields. Thus, developing crop varieties with inbuilt genetic tolerance to environmental stresses is urgently needed. Selective breeding based on genetic diversity is not keeping up with the growing demand for food and feed. However, the emergence of contemporary plant genetic engineering, genome-editing, and synthetic biology offer precise tools for developing crops that can sustain productivity under stress conditions. Here, we summarize the systems biology-level understanding of regulatory pathways involved in perception, signalling, and protective processes activated in response to unfavourable environmental conditions. The potential role of noncoding RNAs in the regulation of abiotic stress responses has also been highlighted. Further, examples of imparting abiotic stress tolerance by genetic engineering are discussed. Additionally, we provide perspectives on the rational design of abiotic stress tolerance through synthetic biology and list various bioparts that can be used to design synthetic gene circuits whose stress-protective functions can be switched on/off in response to environmental cues.

## 1. Introduction

Climate change is constantly altering the environment in which agricultural practices and crops evolved over the years [1]. Plant distribution and production are influenced by abiotic variables, which are natural components of the environment. Environmental conditions, drought, heat, cold, and high soil salinity, are considered abiotic stresses, and they confront crops in field conditions. These abiotic stressors restrict the global use of arable lands and negatively impact agricultural productivity [2]. Global food production must increase by 70% by 2050 to fulfil the demand imposed by the rising global population [3, 4]. Thus, the knowledge of mechanisms involved in plant abiotic stress sensing, signalling, and regulatory processes associated with adapting to stressful circumstances is crucial for global food security.

**Figure 1:**
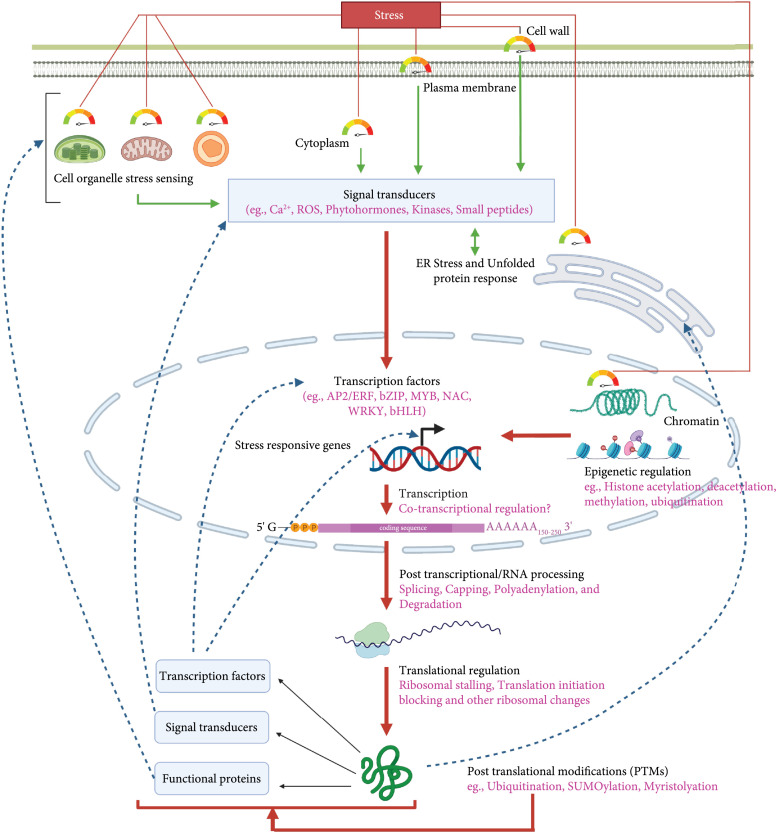
An overview of abiotic stress response sensing, signal transduction, and regulation in plant cells. Plant cells can perceive/sense abiotic stress in several cellular compartments, and the signal transducers (e.g., secondary messengers such as Ca^2+^, ROS, phytohormones, kinases, and signalling (small) peptides) trigger the regulatory pathways involving transcription, posttranscription modifications, translation, posttranslational modifications, and epigenetic regulation. Multiple stress signals activate the stress-responsive transcription factors, which then regulate the stress-inducible gene expression cascade. Some stress-inducible genes code for functional proteins that directly impact role in stress tolerance; others encode regulatory proteins such as signal transducers.

The cascades of regulatory pathways are activated in plants during an abiotic stress response, enabling them to react and adapt to their environment efficiently [5]. Understanding the stress-responsive molecular processes requires a better knowledge of the associated bioparts. Detailed molecular, genetic, and biochemical investigations have highlighted that complex and interconnected molecular networks are involved in stress perception/sensing, signalling, transcription, translation, RNA processing, protein processing, and epigenetic modifications [6] (Figure 1). These molecular responses elicited by different abiotic stresses can be shared or stress-specific [7]. Additionally, cross talk between diverse signalling and regulatory pathways lead to synergetic or antagonistic interactions critical for plant abiotic stress response. A comprehensive understanding of plants’ response to environmental stressors will aid in developing methods for imparting abiotic stress resistance in crops, thereby assuring plant survival and increased productivity.

Traditional breeding strategies are constrained by limited genetic diversity with higher productivity under stress and the finite efficient selection methodology. Using traditional breeding, few varieties have been introduced with enhanced abiotic stress tolerance in field conditions [8]. Genetic modification and engineering techniques are considered more precise and reliable for imparting stress tolerance in crops than conventional approaches [9, 10]. These techniques are centred on endogenous system enhancement by intervening at various phases of the abiotic stress response, including signal transducers, regulatory elements, transcription factors, sensors, effectors, and genes involved in metabolism. However, an abiotic stress response is a multigenic trait, and genetic modification approaches instead regulate individual components [11]. Therefore, there is a requirement for rational and efficient approaches for improving abiotic stress tolerance in crops.

The upstart field of synthetic biology (SynBio) can play a major role in overcoming these complex challenges [12, 13]. Plant synthetic biology is now trailing behind bacterial, yeast, and mammalian systems, where these methods are already altering basic research and the biotechnology industry [14–16]. The standardisation of genetic components and the development of modular cloning techniques in the plant sector were the initial steps towards broader synthetic biology technologies [17, 18]. Synthetic techniques for regulating gene expression and cellular processes, particularly chemically inducible systems, CRISPR/Cas9-based technologies, and other advancements in genome engineering, are critical for advancing plant synthetic biology in the future [12, 19–21].

The effective design of genetic circuits is a prerequisite for producing sentinel plants with desirable characteristics [18]. Plant genetic functions are complicated and influenced by various environmental signals, affecting synthetic gene circuit regulation. The genetic components should be able to act independently of the plant’s endogenous regulatory system. Furthermore, genetic circuits can be activated by external regulation, which potentially assists in switching the desired trait ON/OFF as and when required [22]. Control over synthetic genetic circuits can be further improved by introducing additional regulatory components (e.g., terminators and insulators). Thus, SynBio is a promising tool that can be widely utilised to develop plants with the ability to detect specific, combined, or multiple abiotic stressors displaying enhanced stress tolerance and overall increased crop productivity in the field.

Thus, this chapter will discuss the potential applications of synthetic biology approaches for improving abiotic stress tolerance in crops. In particular, we will focus on the current understanding of the molecular mechanisms involved in the regulation of the major abiotic stresses, namely, heat, cold, drought, and salinity in plants, followed by summarizing the validated and predicted bioparts which can be further explored for improving abiotic stress tolerance in crops by adopting synthetic biology.

## 2. Abiotic Stress Sensing/Perception

**Figure 2:**
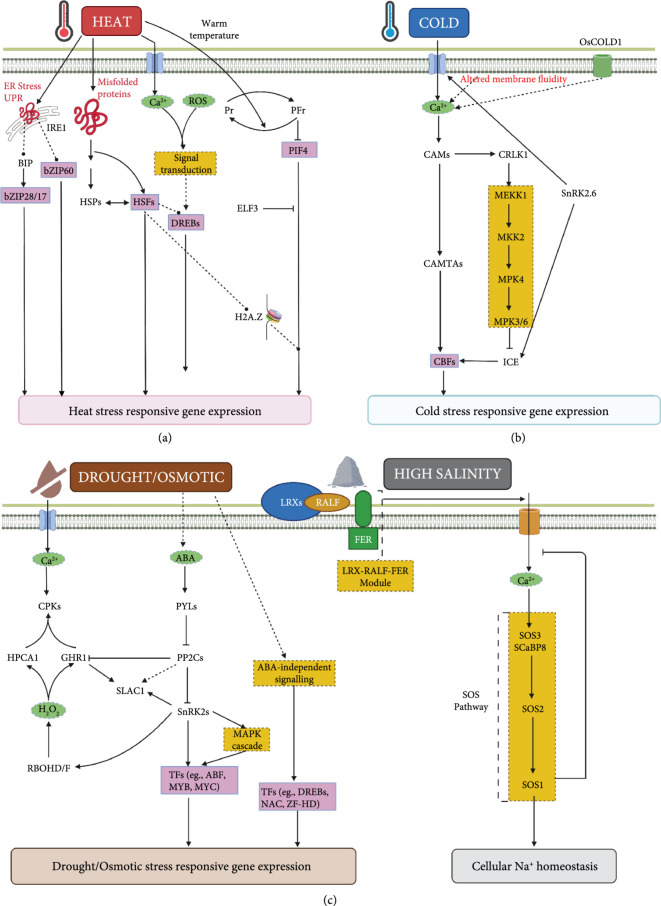
A schematic representation of sensing and signalling cascades associated with various abiotic stresses. (a) Heat: heat induces misfolding of proteins that bind with HEAT SHOCK PROTEINS (HSPs), releasing HEAT SHOCK FACTORS (HSFs), which are then free to mediate heat-responsive gene expression. Misfolded proteins caused by heat stress can also activate the unfolded protein response (UPR) signalling pathway in the endoplasmic reticulum (ER). The ER-associated UPR signalling pathway has two arms, one involving two ER membrane–associated TFs, bZIP17, and bZIP28, and the other involving the RNA-splicing factor IRE1 and its target bZIP60 mRNA. When unfolded proteins attach to the luminal domain of IRE1, they dimerize (or oligomerize) and activate RNase activity, which cleaves bZIP60(u) mRNA, resulting in a spliced form of bZIP60. The spliced variant’s translation creates of active bZIP60 TF protein, which transport to the nucleus activates the stress-responsive genes. When BiP is separated from the ER-anchored transcription factors bZIP28/17, they are mobilised to the Golgi and delivered to the nucleus. bZIP28/17 binds to ER stress response elements in the nucleus to increase the transcription of UPR genes. Phytochrome-mediated signalling may detect warm temperatures. Heat-induced conversion of PhyB from the active Pfr form to the inactive Pr form frees PIF4 from Pfr inhibition, resulting in the activation of heat-responsive genes. When exposed to heat, ELF3 undergoes a phase change and aggregates, losing its capacity to suppress transcription of heat-responsive genes. Heat-induced replacement of H2A.Z by H2A in nucleosomes at specific genes enhances chromatin accessibility for transcription. Heat-induced Ca^2+^ spikes and ROS accumulation detect changes in membrane lipid fluidity. (b) Cold: calcium ion channels may contribute to cold-induced Ca^2+^ spikes by detecting altered membrane fluidity. In rice, OsCOLD1 is required for cold-induced Ca^2+^ increases. Cold stress activates the MEKK1–MKK2–MPK4 module (yellow box), linked to Ca^2+^ signalling via protein–protein interactions between CRLK1 and MEKK1. Additionally, cold induces the release of SnRK2.6, which results in the production of CBFs, which control the transcription of cold-responsive genes. Cold-induced Ca^2+^ signalling can directly activate the CBF regulon via the CAMTAs. (c) Drought and salinity both induce hyperosmotic stress, which is thought to alter the tension of the bilipid membrane, which may be recognised by Ca^2+^ channels leading to the induction of Ca^2+^ spikes. Both ABA-dependent and ABA-independent signalling are initiated in response to hyperosmotic stress. Additionally, stress-induced H2O2 is likely recognised by the Leucine-rich repeat receptor-like kinase (LRR-RLK) gene HPCA1 and, more particularly, by GUARD CELL HYDROGEN PEROXIDE RESISTANT1 (GHR1) in guard cells to produce Ca^2+^ signals via Ca^2+^ channel activation. This Ca^2+^ signal is sent to guard cells by protein kinases CPKs, which phosphorylate ABA-response effectors such as SLOW ANION CHANNEL-ASSOCIATED 1. (SLAC1), potentially enhancing stomatal closure in response to osmotic stress sensing. The signalling network demonstrates the critical functions of protein phosphorylation, calcium signalling, and ABA signalling in response to hyperosmotic stress. Salinity stress degrades the integrity of the cell wall, which may be detected by the LRX–RALF–FER module. Ca^2+^ stimulates the SOS3–SOS2–SOS1 pathway, which is responsible for maintaining cellular Na^+^ homeostasis.

A primary stress sensing/perceiving mechanism translates the abiotic stress stimuli into a biological signal. In plants, the identification of abiotic stress sensors has been a challenging task due to the redundant nature of multiple sensors and the criteria used to define primary sensors. Addressing these limitations, four principal characteristics for defining a stress sensor have been proposed [23, 24]: (1) the true stress sensor must sense the abiotic stress by only perceiving the alterations in the environmental conditions, (2) the structure and activity of the cellular component must be directly altered in response to an abiotic stress stimulus, (3) the alterations in the cellular component must trigger a signal transduction pathway, and (4) the alterations lead to adaptive changes upon abiotic stress exposure. The identification of the abiotic stress sensing mechanisms has been a challenging task. Based on the outcomes of several studies adopting indirect approaches to identify abiotic stress sensors, putative stress sensors can be defined for the major abiotic stresses, namely: temperature, drought, and salt stress (Figure 2).

### 2.1. Perception of Temperature Stress

Plants get exposed to temperature changes that vary in range, intensity, and duration daily and seasonal. Temperature changes affect enzyme kinetics, membrane fluidity, and protein folding makes it challenging to distinguish temperature-induced physiological changes from the actual sensing mechanism [23]. Plants respond in a variety of ways when temperatures rise over optimum levels. In Arabidopsis, exposure to warm ambient temperatures of up to 30°C induces changes in morphology and development known as thermo-morphogenesis, which may help avoid future heat stress [25]. Upon warm heat stress, temperature-dependent switching of phytochrome B (PhyB) from active to inactive state results in inhibition of phyB-mediated repression of the transcription factor PHYTOCHROME INTERACTING FACTOR-4 (PIF4) [26, 27]. This leads to the accumulation of PIF4, which promotes thermo-morphogenesis, such as promoting hypocotyl elongation [28, 29]. The activity of phyB as a thermo-sensor needs light activation [27]; thus, it is hypothesised that a separate and unknown thermo-sensing mechanism occurs in the root system. Recently, it was also proposed that warm ambient temperature sensing involves condensation of EARLY FLOWERING 3 (ELF3), which inhibits the transcriptional binding of ELF3 with its target genes [30]. Since ELF3 acts as a transcriptional repressor, its failure to bind to its target genes promotes their expression. The temperature responsiveness of the ELF3 was attributed to a polyglutamine (poly Q) repeat, entrenched within a prion domain (PrD). Moderate (20-38°C) to severe (>40°C) heat stress results in the accumulation of misfolded proteins, which induces the expression of HEAT SHOCK PROTEINS (HSPs) in an attempt to achieve cellular protein homeostasis [31, 32]. The binding of HSPs to misfolded proteins releases HEAT SHOCK FACTORS (HSFs), which then bind to the heat shock elements (HSEs) of their downstream targets, thereby regulating heat stress-responsive gene expression [33, 34].

Several potential sensors in the cold stress sensing pathway have been postulated, but their role as true cold sensors still requires verification [35]. The decrease in cell membrane fluidity after cold stress is commonly regarded as a key cold sensing mechanism [36]. DIACYLGLYCEROL KINASE (DAGK) activity, which occurs within seconds of cooling exposure, is linked to membrane fluidity [37]. Furthermore, the amount of desaturated fatty acids in the plasma membrane is related to its fluidity and is associated with FATTY ACID DESATURATION2 (FAD2) gene encoding the oleate desaturase. Mutations in FAD2 mutation reduce several physiological responses to cold stress [38]. In mammals, temperatures below optimum levels are sensed by TRANSIENT RECEPTOR POTENTIAL (TRP) ion channels [39, 40]. Ion channels orthologous to TRP are not known in plants. Cold-induced gene expression in plants is Ca^2+^ dependent [41, 42]. As a result, it is conceivable that ion channels (such as Ca^2+^ channels) and electrophysiological responses also play a role in low-temperature sensing in plants.

### 2.2. Perception of Drought Stress

Drought causes osmotic stress in plants; thus, reduction in osmotic potential is likely the earliest sign of water limiting conditions. Even though several basic drought sensors have been postulated, the intricacy of plant responses to water-limiting situations and the presence of potential multiple redundant osmo-sensors make it difficult to identify true osmo-sensors. Turgor loss caused by hyperosmotic stress modifies lateral tension on the bilipid membrane. Research indicates that increasing membrane tension in response to drought stress activates OSCA1, which encodes for a membrane hyperosmolality-gated calcium channel, resulting in the influx of Ca^2+^ ions [43]. In *Arabidopsis*, *osca1* mutants, seedlings were grown under osmotic stress decreased primary root length and leaf area, indicating an enhanced susceptibility to osmotic stress. OSCA1 has a transmembrane domain similar to the Domain of Unknown Function221 (DUF221) domain present in the drought-responsive protein EARLY RESPONSIVE TO DEHYDRATION4 (ERD4; Ganie, Pani [44]). Additionally, CALCIUM PERMEABLE STRESS-GATED CATION CHANNEL1 (CSC1), an OSCA1 homolog (OSCA1.2), is depicted to be involved in osmotic stress sensing [45]. However, the precise function and subcellular localisation of CSC1A in plants are unclear.

### 2.3. Perception of Salt Stress

Upon exposure to salt stress, along with hyperosmotic stress, the plant also experiences ionic stress. While osmotic changes caused by salt stress may be detected using sensing mechanisms similar to those described above for drought stress, a different mechanism would be essential to detect the ionic stress. Ionic stress induces salt stress-specific Ca^2+^ signatures, which were recently investigated to understand the salt sensing mechanisms in plants [46]. It was proposed that Na^+^ might be detected by membrane lipid microdomains containing the sphingolipid Glycosyl Inositol Phosphoryl Ceramide (GIPC), which MOCA1 generates and binds Na^+^, resulting in salt-induced Ca^2+^ spikes. The channels that mediate the Ca^2+^ spikes are unknown; however, ANNEXINS1 (ANN1) and ANN4 are plausible candidates [47, 48].

The salt stress-triggered spike in intracellular Ca^2+^ is perceived by the classical Salt Overlay Sensitive (SOS) pathway [49]. The plant SOS pathway components: SOS3 and SOS3-LIKE CALCIUM BINDING PROTEIN8 (SCaBP8) acting as a Ca^2+^ sensor, SOS2 encoding a serine/threonine kinase and SOS2-LIKE PROTEIN KINASE (PKB5), and SOS1 encoding a plasma membrane Na^+^/H^+^ antiporter [50, 51]. Within a few seconds of salt stress exposure, the Ca^2+^ sensors of the SOS pathway are activated, which in turn activates SOS2. Through direct phosphorylation, the SOS3-SOS2 complex regulates SOS1 expression and function [52]. Salt stress-specific Ca^2+^ signatures regulate the SOS1 activation. The SOS3-SOS2-SOS1 cascade thereby initiates Na^+^ export to maintain cellular Na^+^ homeostasis.

## 3. Signalling Pathways

Stress perception or sensing triggers intricate response machinery involving a well-adjusted orchestration of signalling molecules, transcription factors, metabolic compounds/molecules, and other regulatory molecules. The sessile nature of plants has directed the evolution of highly robust, flexible, and sophisticated signalling networks which either utilise functionally redundant genes or multiple pathways existing and functioning parallelly. Thus, in this section, based on the available research findings, the molecular mechanisms involved in the regulation of abiotic stress signalling will be discussed in detail.

### 3.1. Calcium Signalling

Abiotic stress increases calcium ions into the cytosol beyond the threshold concentration inducing damage to the cell membrane and organelles [53]. Calcium homeostasis in the cell is then regulated by several ion channels, transporters, and intracellular organelles. The fluctuations in calcium concentration are stress/stimuli specific and spatially and temporally discrete signatures [54]. These calcium signatures are decrypted by calcium-binding proteins, namely, CALMODULIN (CaM), CAM-LIKE (CML), CALCINEURIN B-LIKE (CBL), CALCIUM-DEPENDENT PROTEIN KINASE (CDPK/CPK), and CALCIUM- AND CALMODULIN-DEPENDENT PROTEIN KINASE (CCaMK), which then bind to downstream effector molecules [55, 56]. CPKs, CBLs, and CMLs have been identified in protozoans and plants, but CaMs are extensively conserved across all eukaryotes [55]. The proteins associated with calcium signalling have characteristic EF-hands motif with distinct patterns. Most of the abovementioned calcium-binding proteins have four EF-hands, except CBL, which has three [57].

CPKs play a major regulatory function in the Ca^2+^-sensing protein families by binding directly to Ca^2+^ [58]. CDPK phosphorylate downstream protein targets in response to dynamic variations in cytoplasmic Ca^2+^ concentrations induced by hormones and abiotic stressors to control growth and stress responses [59, 60]. The significant role of CPKs in abiotic stress tolerance was validated via loss-of-function and gain-of-function experiments. CPK activity is verified by global expression studies, which reveal that many CPK members demonstrate stress-specific expression. Studies targeting abiotic stress tolerance in crops have identified CPKs as potential candidates [61]. For instance, in rice, drought tolerance was imparted by overexpression of *OsCPK9* [62].

The activation of the SOS pathway exemplifies how Ca^2+^ signatures trigger particular intracellular Ca^2+^ sensing proteins, thereby regulating downstream transcription, translation, and further interactions in response to abiotic stress. Similarly, Ca^2+^ signals are transduced to the calmodulin-binding transcriptional activators Calmodulin-binding transcription activator (CAMTA)—CAMTA1, CAMTA2, and CAMTA3 stimulating CBF genes expression by binding to their promoters and thus mediate cold stress responses [63]. The stress response generated by specific calcium signatures is also governed by the colocalization and timely expression of calcium sensors and their putative partner and downstream proteins.

### 3.2. ROS-Mediated Signalling

Reactive oxygen species—ROS (O_2_^-^, H_2_O_2_, OH radical, and O_2_) —formerly considered as entirely harmful to plant life are produced in nearly all cell components during various enzymatic processes and upon exposure to abiotic stress [64]. Respiratory burst oxidase homologs (RBOHs), peroxidases, and oxalate oxidase are the proteins responsible for most ROS generation [65–67]. Elevated ROS levels are reduced to maintain cellular homoeostasis by the scavenging activity of enzymes, superoxide dismutase (SOD), catalase (CAT), glutathione reductase (GR), glutathione peroxidase (GPX), ascorbate peroxidase (APX), and peroxiredoxin (PRX) [68]. Plants also generate antioxidant compounds such as thiols, ascorbic acid, carotenes, and flavonoids to neutralise excess ROS [69]. ROS act as an effective signalling molecule both at the single-cell and cell-to-cell levels because of the mechanism involved in maintaining a fine balance of ROS in plant cells.

Under osmotic stress, ROS can increase unaided of stress-induced ABA accumulation; however, H_2_O_2_ generation is controlled by abscisic acid (ABA) signalling pathway via SNF1-related protein kinase 2 (SnRK2) and protein kinase-mediated activation of the NADPH oxidases (RbohD and RbohF) [70, 71]. Furthermore, extracellular H_2_O_2_ is likely sensed across the plant by the Leucine-rich repeat receptor-like kinase (LRR-RLK) gene HPCA1 and specifically in guard cells by GUARD CELL HYDROGEN PEROXIDE RESISTANT1 (GHR1) to induce Ca^2+^ signals via Ca^2+^ channel activation. This Ca^2+^ signal is transduced to guard cells by protein kinases CPKs which can phosphorylate ABA-response effectors, including SLOW ANION CHANNEL-ASSOCIATED 1 (SLAC1) [72, 73]. Thus, upon osmotic stress sensing, in addition to the ABA-dependent signalling module (discussed later in the section), stomatal closure is facilitated by an H_2_O_2_ HPCA1/GHR1–Ca^2+^–CPK module.

According to recent research, ROS build-up and Ca^2+^ generation both increase the induction of the other in response to abiotic stress exposure [74, 75]. Superoxide anions generated by RBOHD stimulates Ca^2+^ channels, activating the TWO PORE CHANNEL1 (TPC1, a vacuolar Ca^2+^ channel). TPC1 transfers Ca^2+^ accumulated in the vacuoles, which then activates RBOHD [76]. This feedback loop is potentially crucial for rapidly transmitting of stress-responsive ROS and Ca^2+^ waves (especially during salt stress) [77]. Abiotic stress such as drought and heat produces similar Ca^2+^ and ROS signatures across the plasma membrane [74, 75]; however, the elaborative mechanism is unclear.

### 3.3. Protein Kinase-Mediated Signalling

In eukaryotes and prokaryotes, protein phosphorylation acts as a ubiquitous signalling pathway. Protein kinases are divided into many groups based on their ability to phosphorylate specific amino acid residues. Experimentally validated two-component system (TCS) comprising of a histidine kinase (HK; signal sensor) and a nuclear effector response regulator (RR; transcription factor); play key roles in abiotic stress-induced signalling via a phosphorylation process [78]. The phosphoryl group is transferred from a conserved histidine (His) residue on the HK to a conserved aspartate (Asp) residue on the RR in the sensor-regulator coupling process between these two components [79]. There are also sophisticated TCSs with a multistep His-Asp phosphorelay in plants potentially providing an additional regulatory checkpoint.

The mitogen-activated protein kinase (MAPK) module is also part of the protein kinase family and is triggered by various stimuli including mitogens, phytohormones, and environmental stressors [80–82]. A typical MAPK module comprises three protein kinases that activate each other via relay phosphorylation. These protein kinases are a MAP kinase kinase kinase (MKKK or MEKK), a MAP kinase kinase (MKK or MEK), and a MAP kinase (MAPK or MPK). An active MEKK activates downstream MKK via phosphorylating two serine and/or threonine residues in its activation loop (S/T-X3 5-S/T) [83]. MKK activation leads to dual phosphorylation of a conserved motif, T-X-Y, in the activation loop of MAPK, thereby activating it. The activated MAPK then phosphorylates and changes the activity of the downstream target, allowing for downstream reactions [84].

### 3.4. Phytohormone-Mediated Signalling

Phytohormones are generated in extremely low quantities yet can control a wide range of cellular activities in plants [85]. They function as chemical messengers in higher plants, communicating cellular processes, and therefore, they perform critical functions in the abiotic-stress response, coordinating different signal transduction pathways [86]. Their essential functions of facilitating plant acclimation to the environments through plant growth, development, and nutrient allocation are thoroughly appreciated [87].

ABA, a key phytohormone, plays a vital role in regulating the abiotic stress response. It also functions in developmental processes like seed germination, seed dormancy, stomatal closure, and flowering [88–91]. In plants, the ABA signal transduction involves ABA receptors (PYR/PYL/RCAR), SnRK2 kinases (positive regulators), and type 2C protein phosphatases (PP2C) [92–95]. Under the lack of ABA conditions, PP2Cs bind to SnRK2s and block them from activating. Because inactive SnRK2s cannot phosphorylate downstream substrates, signal transduction does not proceed. In the presence of ABA, PYR/PYL/RCAR receptors bind to ABA and, through the interaction with PP2Cs, release SnRK2s. Autophosphorylation of the activation loop then activates the SnRK2s. The activated SnRK2s can phosphorylate substrate proteins such as ion channels, transcription factors, and enzymes (NADPH oxidases), triggering ABA responses. Other protein kinases control the activity of SnRK2s. SnRK2 may be activated by a Raf-like kinase (B3-MAPKKK) by activation loop phosphorylation; however, casein kinase 2 (CK2) can phosphorylate SnRK2’s carboxyl-terminal serine residues, increasing SnRK2-PP2C binding and resulting in inactivating SnRK2 [96]. ABA acts as a promoter of abiotic stress tolerance [97]. Exogenous administration of ABA or synthetic ABA mimics (i.e. ABA receptor agonists) is reported to elicit a stress response in plants, which improves their adaptability, showing the relevance of its activity under stress circumstances [98, 99].

Plants are also reported to produce ethylene in response to a variety of environmental stressors. Ethylene biosynthesis involves two steps. The first step is the transition of S-adenosyl-L-methionine (SAM) into 1-aminocyclopropane-1-carboxylic acid (ACC) via ACC-Synthase. In contrast, the second step involves the conversion of AAC to ethylene catalysed by ACC oxidase (ACO) [100]. Ethylene activates ER-located membrane protein ETHYLENE INSENSITIVE 2 (EIN2), which targets EIN3-BINDING F-BOX 1 (EBF1) mRNA to the cytoplasmic processing body (P-body) [101]. EIN2-mediated ethylene signalling also leads to translational inhibition of F-box binding proteins, EBF1, and EBF2 [101]. The function of ethylene as a signalling molecule is influenced by reactive oxygen species (ROS) quantity. Previous findings that *ein2* and *etr1* mutants had poor basal thermotolerance [102] and freezing tolerance [103], and ectopic overexpression of ERF74 improved heat tolerance and other abiotic stress tolerance [104], offer evidence that ethylene plays a significant role in abiotic stress response. Recently, EIN3-ERF95/ERF97-HSFA2 transcriptional cascade was shown to play an essential role in regulating basal thermotolerance and heat stress-responsive gene expression in plants [105].

Another phytohormone class, brassinosteroids (BRs) plays many plant growth and development roles. Plant-specific BR ligands bind directly to the membrane-bound LRR-RLK, BRASSINOSTEROID INSENSITIVE 1 (BRI1), and BRI1 ASSOCIATED RECEPTOR KINASE (BAK1), triggering signalling through cytoplasmic phosphorylation cascades including phosphorylation of serine /threonine phosphatase protein (BSU1) protein and proteasomal destruction of BIN2 (BRASSINOSTEROID INSENSITIVE 2) protein kinases [106–108]. Inactivation of BIN2 allows BRI1 EMS SUPPRESSOR1 (BES1) and BRASSINAZOLE-RESISTANT 1 (BZR1) to gain entry in the nucleus and activate the expression of target genes [109]. BR interacts with other phytohormones in all of these signalling pathways. Plant growth and survival in drought stress are regulated by BR signalling via BIN2, which interacts with the autophagy system [110]. BR-pretreatment triggers the synthesis of ethylene under salinity [111], and therefore, signalling is increased by increasing the production of 1-ACS [112]. Upon exposure to high salinity, BR exogenous application also enhances the expression of ethylene signalling genes in cucumber, canola, and wheat [113–115]. Furthermore, the BR signal promotes ROS generation by NADPH oxidase, which activates MAPKs, causes protein phosphorylation, and targets genes involved in cellular defence [116].

Cytokinins are reported to perform a critical and multifaceted role in abiotic stress response. At the plasma membrane and ER, HISTIDINE KINASES (AHK2, AHK3, and AHK4/CRE1/WOL) detect cytokinins [117]. Recently, a small proportion of plasma membrane located AHKs can mediate the extracellular cytokinin signal has been reported [118]. Cytokinins bind to the CHASE domain of the receptor and stimulate intracellular histidine kinase (HK) activity, which leads to sensor autophosphorylation [119]. Cytokinin is often thought to regulate plant stress response negatively; however, this is not always firmly substantiated. Transgenic tobacco plants expressing the isopentenyl transferase (IPT, sourced from *Agrobacterium tumfaciens*), preceded by a stress-inducible promoter, showed improved tolerance to water-deficit conditions due to boosted cytokinin levels [120]. These findings were reproduced in transgenic rice [121] and peanut [122] plants utilising the same stress-induced cytokinin circuit. However, in contrast to the above findings, *Arabidopsis ipt* mutants with lower cytokinin levels are drought tolerant than the wild type [123]. Similarly, reduced cytokinin levels, obtained by constitutive or root-specific overproduction of cytokinin oxidase (CKX), the cytokinin-degrading enzyme, have a beneficial effect on drought tolerance [124, 125]. Furthermore, heat stress regulates the expression of several CK responsive genes [126], and exogenous cytokinins enhance plant heat tolerance [127]. The reduction of photosynthesis and chloroplast growth caused by heat stress is alleviated by exogenous administration of cytokinins and increased endogenous cytokinin levels.

Other phytohormones also perform regulatory roles in plants’ abiotic stress response [128, 129]. Salicylic acid (SA) is linked to the control of a variety of physiological activities, including photosynthesis, the formation of the antioxidant glycine betaine, proline metabolism, the plant-water relationship during stressful situations, and stress tolerance against abiotic stressors. The accumulation of SA causes reduced plant development, which reduces plant fitness. In response to stress, SA signalling is also reported to be linked with the accumulation of ROS. Similarly, gibberellins (GA) are phytohormones that control cell division and elongation, making them necessary for plant growth and development [130]. They also govern cellular redox equilibrium, which is essential in stress signalling via ROS signalling pathways. One of the most significant components involved in stress signalling is the DELLA protein which negatively regulates GA signalling [131]. The DELLA protein controls the production of ROS-scavenging proteins in plants, preventing oxidative damage and extending plant life and fitness [132, 133]. Another class of phytohormones—jasmonic acid (JAs) and methyl jasmonates (MeJAs)—have also been linked to a variety of physiological functions, including abiotic stress response [134]. Exogenous administration of JAs has been shown to improve plant stress resistance when tested on several plants under abiotic stressors such as salt, drought, and temperature (low/high) conditions.

### 3.5. G-Protein Coupled Receptors Mediated Signalling

The G protein (guanine nucleotide-binding protein) coupled receptors signalling module includes the G*α*, G*β*, and G*γ* subunits and is an evolutionarily conserved extracellular signal route [135]. In humans, the G-protein complex comprises 23 G*α*, five G*β*, and 14 G*γ* subunits [136]. Plant G proteins, on the other, include only one G*α* subunit, three different G*α*-like subunits (XLGs), one G*β*, and varying numbers of G*γ* subunits depending on species [137]. Plant heterotrimeric G protein signal transduction pathway differs from animals. In contrast to animal G proteins, plant G proteins can self-activate without the help of GPCRs (G-protein-coupled receptors). For instance, G*α* protein AtGPA1 can exchange GDP with GTP without the need for a GPCR, thereby activating it [138]. However, GTPase activity-accelerating proteins (GAPs) are involved in hydrolysing GTP and deactivating the G*α* protein, AtGPA1 [139]. Additionally, activation of G*α* or atypical G*α* -like subunits in plants is ineffective in dissociating the G protein heterotrimer.

Studies have identified K^+^ and Ca^2+^ channels as key downstream effectors of heterotrimeric G protein. For instance, when plants are exposed to low temperatures, COLD1 interacts with a subunit of G protein to activate Ca^2+^ channels and boost G protein’s GTPase activity; in turn regulating the transcriptional expression of several stress-related genes, including OsAP2, OsDREB1A, OsDREB1B, and OsDREB1C [140]. Under drought stress, G*β* subunits are reported to upregulate NCED gene expression, favourably regulates ABA production. ABA-responsive genes (e.g., AtMPK6, AtVIP1, and AtMYB44) in *agb1-2 Arabidopsis* mutants are significantly upregulated after ABA or drought treatment [141]. Another subunit of the G-Protein module; G*α* controls plant responses to salt stress potential by either attenuating cell cycle regulation in response to hyperosmotic stress or regulating cellular senescence in response to ionic stress [142].

Plant G protein activation/deactivation mechanisms are unclear, as are their direct effectors and connections with different transcriptional or protein networks. When comparing various plant lineages, there is also variation in the components and mechanisms of action. Thus, further studies targeting crop species are required to understand the G Proteins mediated abiotic stress signal transduction fully.

### 3.6. Signalling Peptides

Signalling peptides are short 5-10 or 40-100 amino acid long peptides, recently identified as abiotic stress-responsive signalling molecules [143, 144]. A major class of plant signalling peptides, CLAVATA3(CLV)/EMBRYO-SURROUNDING REGION RELATED (CLE) peptides, are ~12–14 amino acids long [145]. In *Arabidopsis*, CLE25 and CLE9 are involved in drought stress response [146, 147]. CLE25 is a transportable peptide that connects dehydration stress tolerance to abscisic acid- (ABA-) mediated tolerance by plausibly transmitting dehydration signals via CLE25–BAM modules from the roots to the leaves. This module acts via long-distance signalling to increase ABA accumulation by upregulating NCED3 expression [146]. By controlling stomatal closure, CLE9 helps to improve drought resistance by potentially interacting with the OST1 and anion channel protein SLAC1 protein [147]. In *Arabidopsis*, another member of this class, CLE-45, associated with the CLE45-STERILITY-REGULATING KINASE MEMBER1 (SKM1)/SMK2 receptor module promotes pollen tube development and results in effective seed setting in response to heat stress response in plants [148].

Another class of signalling peptides, RALF peptides, are 5 kDa cysteine-rich peptides involved in salt stress signalling [149]. The module involving LRX, FERONIA (FER), and RALF in *Arabidopsis* is suggested to detect high salinity-induced cell wall defects. LRX3/4/5 proteins have been found to bind with the peptide ligands RALF22 and RALF23, blocking their interaction with FER, a plasma membrane-localized receptor-like kinase (RLK) that potentially interacts with cell wall pectins. Salt stress disrupts these connections, leading to FER-dependent Ca^2+^ surge in the early elongation zone of roots [150]. The mechanism of how salt stress influences the interaction of LRXs with cell wall pectin and RALFs requires to be validated through biochemical experiments. Although few other signalling peptides have been discovered to coordinate plant abiotic stress, the molecular processes of this peptide signalling still need to be elucidated in detail.

## 4. Metabolic Pathways

Plants respond to diverse abiotic stimuli in different ways, and one of the most prevalent reactions is alterations in primary metabolism. ROS accumulation occurs under abiotic stress due to a disruption in PSII's electron transport chain [151]. Accumulation of ROS harms cells by causing membrane lipid peroxidation, and thus, plants have developed various methods to regulate lipid peroxidation, including the production/accumulation of numerous metabolites [152]. Similarly, the levels of secondary metabolites are also regulated in response to abiotic stress [153], but these changes are species- and stress-dependant.

### 4.1. Carbohydrate Metabolism

Plants are both producers and consumers of carbohydrate molecules. Photosynthesis produces a variety of sugars to maintain plant growth and development. They are essential regulators of abiotic stress responses in the cell, and their well-known function in numerous physiological processes. Tolerance to different environmental stresses is conferred by accumulating of soluble sugar molecules and sugar polyols and different levels of starch-sugar interconversion [154, 155]. These molecules stabilise cellular integrity (structure and osmotic potential) by serving as an osmolyte/osmoprotectant. These molecules also get interlinked into stress signalling pathways and assist in maintaining redox equilibrium [156, 157].

#### 4.1.1. Sugar Metabolism

Sugars are the main products of photosynthesis, and they help plants grow and develop by providing energy or synthesising storage and structural components. Adverse environmental circumstances cause differential expression of genes involved in several processes such as photosynthesis, respiration, starch-sucrose metabolism, and cell cycle control, resulting in optimum carbon and energy use. The primary glucose sensor, HEXOKINASE 1 (HXK1), reacts to glucose concentrations under stress and regulates gene expression appropriately [158]. Because invertases are intimately linked to abiotic stress tolerance, glucose derived from invertase activity keeps HXK active, therefore, maintaining mitochondrial ROS equilibrium [156]. In plants, the HXK-independent glucose-sensing pathway has been documented; however, it is not well understood. Furthermore, some plants have fructokinases, which may play stress-induced sugar sensing [159]. Another sugar molecule, trehalose, which is present in low quantities in plants, show elevated levels upon abiotic stress exposure [160]. Endogenous trehalose levels are critical for maintaining development under stressful conditions. When given exogenously in small doses, trehalose reduces physiological and biochemical abnormalities caused by different abiotic stressors in plants by plausibly mediating ROS homeostasis and upregulating the stress-responsive genes in plants.

SnRK1 is another key mediator of stress signalling in abiotic stress reactions leading to the build-up of protective metabolites and defensive chemicals [161]. Stress can cause sugar imbalances, leading to ABA build-up and the activation of a special sugar signalling system. ABI4 is a key ABA sugar signalling downstream effector that regulates sugar sensitive gene expression. ABI4 also promotes the production of ANAC060, which inhibits the ABA signalling pathway in sucrose [162]. Carbohydrates like glucose and sucrose also influence auxin signalling and biosynthesis. The disaccharide sucrose interacts with the GA signalling system by stabilising the DELLA proteins, a negative regulator of GA signalling [131, 162].

#### 4.1.2. Starch Metabolism

In response to abiotic stress, starch metabolism regulation can increase cellular carbohydrates or increase starch storage. Starch breakdown releases a range of sugars upon stress exposure, thereby boosting carbon flow into the hexose phosphate pool in a species-, tissue-, and stress-dependent manner [155]. In spinach, barley, and rice leaves, drought stress has been shown to suppress starch production and increase sugars [163–165]. Drought can cause starch-degrading enzymes to become active, increasing in sugars. Similarly, starch degradation is known to be triggered by cold stress [166]. Cold activation of certain *β*-amylase (BMY) isoforms has been frequently demonstrated based on expression and functional investigations [167]. In cereals, a mild drought postanthesis can activate important sucrose to starch conversion pathway associated enzymes, including Sucrose synthase (SuS), Starch branching enzymes (SBE), and AGPase [168]. Upon salinity stress exposure, a salt-tolerant rice cultivar, “Pokkali,” stored more starch in leaves than the sensitive cultivars examined, allowing the tolerant genotype to maintain photosynthesis [169]. In tomatoes, a similar effect was reported [170]. Furthermore, heat-tolerant tomato cultivars retained pollen starch content upon heat stress exposure, resulting in increased fertility in contrast to sensitive genotypes [171]. However, heat negatively regulates the activity of starch enzymes as the stress proceeds, resulting in a decrease in starch content. Thus, the starch-sugar interconversion in source and sink tissues plays a critical regulatory role in abiotic stress response. However, the current understanding of stress-induced carbohydrate alterations and the process behind these changes remains inadequate.

### 4.2. Amino Acid Metabolism

In plants subjected to abiotic stress, a general build-up of free amino acids has been documented [172]. Autophagy and ABA-triggered protein turnover may potentially lead to this rise in free amino acids levels. Plants can utilise amino acids as an alternate substrate for mitochondrial respiration in instances where there is a lack of glucose supply owing to a drop in photosynthesis rates in response to stress exposure. Plant fitness and, as a result, crop output is potentially affected by not just metabolic adaptations to stress but also by the proficiency of continuing growth processes.

Proline is the most prevalent water-soluble amino acid, and its metabolism in plants has been researched extensively in abiotic stress response. Proline accumulation can rise several folds under abiotic stress compared to nonstressed plants, indicating its involvement in abiotic stress regulation [173]. However, it is still unknown why proline accumulates during stressful situations. Proline has been found to accumulate in the cytosol in response to hyperosmotic stressors, suggesting that it can act as a suitable osmolyte, aiding plants in maintaining an optimal water balance [174]. Proline is also important for maintaining redox equilibrium in plants and preserving cellular integrity [175].

Another essential and effective solute is glycine betaine (GB). By maintaining an appropriate osmotic equilibrium, GB protects cells against the consequences of different stressors [176]. GB also helps to keep the quaternary structure of proteins stable. GB biosynthesis for stress tolerance induction is species/cultivar specific. Under diverse stressors, GB has a variety of protective benefits that are mediated by distinct metabolic processes. A considerable increase in GB accumulation was linked to the preservation of photosynthetic pigments and other biochemical characteristics that were beneficial in maintaining improved development in maize plants grown under osmotic stress [177].

### 4.3. Phenylpropanoid Metabolism

One of the most well-studied secondary metabolic pathways is the phenylpropanoid pathway [178]. The phenylpropanoid pathway involves enzymatic reactions: phenylalanine ammonia-lyase (PAL) catalyse phenylalanine deamination to trans-cinnamic acid, trans-cinnamic acid hydroxylation to 4-coumarate by cinnamic acid 4-hydroxylase (C4H) activity, and 4-coumarate conversion to 4-coumaroyl-CoA by 4-coumarate-CoA ligase (4CL). Various offshoots exist downstream of the main phenylpropanoid route, with the lignin and flavonoid pathways being two of the most important. Lignin deposition aids cell wall thickening during drought stress, allowing plants to retain cell turgor even under drought conditions. Upregulation of genes involved in lignin production (CAD, C4H, C3H, HCT, F5H, 4CL, CCR, COMT, and CCoAOMT) lead to the build-up of lignin, the secondary cell wall thickening, and thereby improving salt, cold, and drought stress tolerance in several plant species [179, 180]. Flavonoids operate as antioxidants, reducing the oxidative damage produced by ROS, which is triggered by abiotic stressors [181, 182]. In rice [183] and tobacco [184], treatment with flavonoids reduces oxidative damage and improves tolerance to salt and drought stress. Additionally, in rice [185], canola [186], and tobacco [184], flavonoid structural gene (CHS and DFR) overexpression enhances anthocyanins, and intermediate flavanol species production decreases ROS generation, thereby conferring salt stress tolerance. Furthermore, overexpression of F3H and DFR resulted in increased drought tolerance in alfalfa [187] and *Arabidopsis* [188]. Flavanols are also crucial for maintaining redox homeostasis and also enhancing pollen tube development and integrity during high-temperature exposure [189].

## 5. Regulatory Pathways

### 5.1. Transcriptional Regulation

The perception of abiotic stress and the signalling cascade that follows leads to the reprogramming of genome-wide transcription. Additional defensive strategies, such as osmotic adjustment, detoxification, repair of stress-induced damage, and attenuation of stress signalling, are triggered by the regulation of stress-responsive genes. Transcription factors belonging to the bZIP, bHLH, MYB, NAC, AP2/ERF, and WRKY families link stress-specific gene expression to upstream signalling [190]. A common strategy for imparting or improving abiotic stress tolerance in crops is to manipulate the expression of TFs genetically.

#### 5.1.1. bZIP TFs

The bZIP TFs, one of the largest and evolutionary conserved TF family, can efficiently activate downstream gene expression upon abiotic stress exposure. These TFs are characterised by the bZIP domain comprising a basic domain and a leucine zipper domain [191]. The highly conserved DNA binding-basic region contains an invariant N-X7-R/K-X9 motif that usually binds to particular ACGT core nucleotide sequences such as A-box, C-box, G-box, and ABRE-elements. The basic region, site of a nuclear localization signal is composed of ∼16 amino acid residues. On the other hand, the less conserved leucine zipper domain comprises heptad repetitions of Leu or other hydrophobic amino acids that play a key role in dimerization and specific DNA sequence recognition. The role of bZIP TFs in stress-specific transcriptional regulation has been established through genetic screening studies in *Arabidopsis*. AtbZIP17 acts as a positive regulator of the salinity stress response by activating the expression of the salt stress-responsive genes ATHB-7 and SES1 [192], while AtbZIP24 was a negative regulator [193]. Furthermore, *Arabidopsis* salt tolerance is negatively controlled by AtbZIP62, which inhibits the transcription of SOS pathway genes [194].

bZIPs have been extensively studied in several crops, and they have been targeted using transgenic methods for imparting abiotic stress tolerance in crops. Overexpression of *GmbZIP2* improved soybean tolerance to drought and salt stress by increasing stress-responsive genes (*GmMYB48*, *GmWD40*, *GmDHN15*, *GmGST1*, and *GmLEA*) expression [195]. In rice, *OsbZIP05/OSBZ8* showed a higher transcription level in salt-tolerant cultivars than sensitive cultivars, suggesting a beneficial role of *OsbZIP05/OSBZ8* in response to abiotic stress conditions [196]. Similarly, in response to drought stress, OsbZIP71 activates transcription of *OsNHX1* and *COR413-TM1* through binding to their promoters. The increased expression of these genes enhances drought tolerance in transgenic rice [197]. bZIP TFs can also regulate stress response by the regulation of plant metabolites. For instance, in soybean, GmbZIP44, GmbZIP62, and GmbZIP78 TFs, activate downstream genes *ERF5*, *KIN1*, *CORl5A*, and *COR78* expression to control and stimulate the synthesis of proline which potentially enhances cold stress tolerance [198].

A small number of bZIP TF family members are also considered vital genes in UPR and the ER upon stress exposure. Plant cells have two subdivisions of the UPR signalling pathway: one comprises two ER membrane-associated TFs -bZIP17 and bZIP28, and the other involves the RNA-splicing factor IRE1 and its target bZIP60 mRNA [199, 200]. In one ER stress responsive UPR pathway, BiP (chaperone) is recruited to aid folding and protection of unfolded proteins, resulting in its separation from bZIP28. Two proteases cleave bZIP28 once it is transported to Golgi bodies. The cytosolic component of the protein is released as a result of this processing, and it subsequently translocates to the nucleus to activate downstream genes. Thus, bZIP28 acts both as a sensor and a signal transducer. Salinity stress activates bZIP17, which enhances the transcription of genes involved in salt stress tolerance and response [192]. In the other ER stress-responsive, UPR pathway IRE spliced the transmembrane domain of *bZIP60*. The spliced *bZIP60* mRNA encodes a nucleus localized protein and induces UPR-related genes transcription. Recently, in maize, bZIP60 was reported to activate the production of an array of HSPs, thereby acting as a key connection between the UPR in the ER in addition to the nuclear/cytoplasmic heat shock system [201].

#### 5.1.2. WRKY TFs

WRKY TFs, one of the largest plant-specific TF families [202], have a characteristic N-terminus located DNA-binding Domain (DBD) with an invariant heptad WRKYGQK motif and a C-terminus located zinc-binding motif. In the abiotic stress response, the various members of the WRKY TF family either interact with the ABA signalling pathway or ROS signalling pathway or act autonomously [203]. In tomatoes, *SlWRKY81* improves drought tolerance by reducing H_2_O_2_ build-up and thus acting as a negative regulator of stomatal closure [204]. WRKY TFs usually regulate the expression of the target genes through their binding to the W-box cis-regulatory element [(T)TGAC(C/T)] to establish cellular homoeostasis. For instance, *SbWRKY30* in sorghum, for example, controls the drought-responsive gene *SbRD19* by binding to the W-box cis-elements and thereby protects plant cells from ROS-induced damage [205].

Functional characterisation of WRKY TF family members in different crop species highlights their potential role in regulating tolerance to single, combined, or multiple abiotic stress. *GmWRKY49* expression was found to be different in salt-tolerant v/s salt-susceptible soybean genotypes [206]. Overexpressing *GmWRKY49* in soybean and *Arabidopsis* conferred improved resistance to salt stress, with enhanced germination rate, survival rate, root length, and proline content. Further, in cucumbers, cold tolerance was enhanced by overexpressing *WRKY46*, which modulated the cold signalling system in an ABA-dependent manner [207]. Furthermore, transgenic rice expressing *OsWRKY11* driven by the *HSP101* promoter showed heat and drought tolerance [208].

#### 5.1.3. MYB TFs

The largest TF family in plants is the MYB TFs, which are characterised by a conserved N-terminal MYB DNA-binding domain (DBD) repeat [209]. Each repetition (Rs) is made up of 52 amino acid residues folded into three -helices (R1, R2, R3), resulting a helix-turn-helix (HTH) structure. MYB transcription factors have one to four DNA-binding repeats in plants. The MYB TF family is classified into- R1-, R2R3-, R1R2R3-, and 4R-MYB TFs based on the position and number of repeats. The bulk of MYB proteins is members of the R2R3–MYB subfamily [210]. MYB transcription factors have been researched extensively and have been shown to regulate the production of secondary metabolites in plants. MYB proteins also perform various functions in the transcriptional regulation of abiotic stress response [211]. However, the regulation mechanism of MYB proteins upon abiotic stress exposure is yet unclear.

Functional characterisation studies have elucidated MYB TF to be potential candidates for imparting abiotic stress tolerance in crops. In *Arabidopsis*, *AtMYB44* overexpression improves drought tolerance by increasing ABA sensitivity and ABA-induced stomatal closure, whereas *atmyb44* knockout plants showed higher sensitivity to drought stress [212]. Furthermore, overexpression of *AtMYB96* led to improved drought resistance by activating cuticular wax production, which prevented leaf surface water loss [213, 214]. Similar cuticular wax accumulation-based enhancement of drought tolerance observed in *Camelina sativa* plants are showing heterologous overexpression of *AtMYB96* [215]. MYBs also have a role in salt stress response. Salt stress increases the expression of *AtMYB20*, and transgenic plants overexpressing *AtMYB20* exhibited better salt tolerance. Suppression of *AtMYB20*, on the other hand, led to hypersensitivity to salt stress [216]. Furthermore, in response to heat stress, MYB30 inhibits the expression of *ANN1* and *ANN4* through binding directly to their promoters [217]. ANNs encode membrane Ca^2+^ transporter proteins that modulate cytosolic calcium signatures, and therefore, the regulation of ANN by MYB30 controls calcium signalling.

#### 5.1.4. AP2/ERF TF

APETALA2/ETHYLENE RESPONSIVE FACTOR (AP2/ERF) TFs have emerged as key regulators of abiotic stress responses [218]. The distinguishing feature of these TFs is the presence of the APETALA2 (AP2)/Ethylene Responsive Element Binding Factor (EREB) DNA-binding domain comprising a conserved domain of 40–70 amino acids. APETALA2 (AP2), RELATED TO ABSCISIC ACID INSENSITIVE 3/VIVIPAROUS 1 (RAV), DEHYDRATION-RESPONSIVE ELEMENT BINDING proteins (DREBs) (subgroup A1–A6), and ETHYLENE RESPONSIVE FACTORS (ERFs) are the four main subfamilies of AP2/ERFs (subgroup V-X).

DREBs detect Dehydration-Responsive or C-Repeat Element (DRE/CRT) on stress-responsive genes with the A/GCCGAC core sequence to impart resistance to drought, cold, and heat abiotic stressors [219, 220]. Overexpression of DREB1s improves *Arabidopsis* plant tolerance to freezing stress. Drought and heat induce DREB2s, which upregulate the expression of DRE-containing drought-responsive genes, LEAs and heat-responsive genes, and heat chaperones [221]. Furthermore, members of the DREB-A4 family, e.g., HARDY (HRD), and the DREB-A6 family, e.g., ERF53, TG/RAP2.4A, and RAP2.4, favourably regulate salt and drought tolerance [222]. *HRD* overexpression in *Arabidopsis* or rice enhanced plant drought and salt tolerance dramatically [223]. DREBs are thought to control response to abiotic stress in plants through an ABA-independent mechanism. However, mounting data indicates that ABA-dependent stress responses are mediated via a number of stress-responsive AP2/ERFs. Furthermore, the AP2/ERF transcription factor RAV1 controls ABA sensitivity by directly interacting with SnRK2s, the essential kinases governing the ABF activity [96]. AP2/ERFs potentially regulate hormone sensitivity and gene expression by collaborating or antagonistically interacting with different hormone signalling components.

#### 5.1.5. bHLH TFs

The bHLH family, extensively found in plants, is the second-largest TF family after the MYBs [224] are characterised by the occurrence of the bHLH domain comprising a DNA-binding N-terminal stretch of amino acids and HLH (helix loop helix) domain required for dimerization. More than half of the plant bHLHs identified contain a conserved HER motif (His5-Glu9-Arg13) which regulates DNA binding and transcriptional control of downstream genes. Although binding selectivity varies, bHLH TFs usually bind with E-box sequences (CANNTG), such as the G-box (CACGTG) cis-elements.

Abiotic stress response and tolerance regulation by bHLH TFs are highly conserved in plants [225]. The bHLH TFs control plant drought tolerance primarily via modulation of ABA sensitivity or regulation of stomata, leaf trichomes, and root hair production. ZmPTF1 promotes root growth and ABA synthesis in maize, which controls drought tolerance [226]. Controlling ROS balance through direct regulation of the expression of a few peroxidase genes is the significant way bHLH TFs contribute to salt tolerance. To improve *Arabidopsis*’ tolerance to salt stress, *AtbHLH112* enhanced the expression of the *POD* and *SOD* genes while simultaneously decreasing the *P5CDH* and *ProDH* gene expression [227]. Another path for bHLH based enhancement of plant salt tolerance is through controlling the accumulation of secondary metabolites. A MAPK cascade regulates *AtMYC2* in response to salt stress which binds the *P5CS1* gene promoter (P5CS1 enzyme is the rate-limiting in proline biosynthesis). The promoter binding activates *P5CS1* leading to enhanced proline biosynthesis and improved salt tolerance [228]. *bHLH* genes are also involved in plant cold tolerance, linked to increased proline accumulation, lower malondialdehyde levels, and less electrolyte leakage. AtICE1/AtbHLH116 interacts with the CBF promoter in *Arabidopsis* at low temperatures, affecting transcription, and the transgenic plants overexpressing AtICE1/AtbHLH116 exhibited increased cold tolerance [229]. Rice OrbHLH001, a homolog of ICE1, may improve transgenic *Arabidopsis* freezing stress resistance [230]. However, OrbHLH001, on the other hand, has a distinct function from ICE1 and is not reliant on the CBF/DREB1 cold-response pathway.

#### 5.1.6. NAC TFs

NAC TF family name, NAC, comes from three genes (No Apical Meristem: NAM, *Arabidopsis* Transcription Activation Factor: ATAF, and Cup-Shaped Cotyledon: CUC), where the NAC domain was discovered for the first time [231]. The N-terminal DNA binding region of NAC transcription factors has a conserved NAC domain, while the C-terminal DNA binding region contains a regulatory domain. The C terminal region directs the interaction of NACs with diverse targets, including but not limited to lipoxygenase, *DEAD/DEAH box helicase*, *PME* or *PMEIs*, and *Homeobox-related* genes.

Across plant species, stress-responsive NACs function in a conserved manner. Abiotic stress activates the production of OsNAC5, OsNAC9, and OsNAC10, and overexpression of these TFs enhanced drought tolerance substantially [232–234]. Additionally, under stress circumstances, transgenic rice plants overexpressing OsNACs showed higher grain yields than wild-type control plants. In tomato, increased abiotic stress tolerance was observed plants with heterologous overexpression of *Arabidopsis ANAC042*/*AtJUB1* [235, 236]. Furthermore, NACs potentially work in tandem with JA and ABA to regulate responses and tolerance to abiotic stress in plants. For instance, in *Arabidopsis*, ANAC096 regulates osmotic stress and dehydration responses by directly interacting with ABF2 and ABF4, key TFs of ABA signalling [237].

### 5.2. RNA Processing (Co- and Posttranscriptional Regulation)

RNA processing pathways such as splicing, capping, polyadenylation, and degradation are central to plant stress responses. Protein components, such as core spliceosomal proteins, proteins involved in spliceosome assembly, and splicing regulators, are largely conserved in plants. The failure of various elements of the RNA processing pathways is reported to significantly impair resistance to abiotic stresses while having no substantial impacts on plant function under stress-free conditions [238].

The spliceosome, a massive macromolecular complex of five ribonucleoprotein subcomplexes, removes introns during splicing (U snRNPs). U1snRNP-associated proteins, including U1-A and LUC7 zinc finger proteins, are required for abiotic stress tolerance. *Arabidopsis* mutants for spliceosomal protein U1A showed a salt stress hypersensitive phenotype *in vitro* and soil and increased in salt stress-induced reactive oxygen species (ROS) accumulation compared with wild-type. This mutant presented splicing defects associated with 5${}^{\prime }$ SS recognition and transcripts encoding ROS detoxification enzymes, such as CSD1 and ACO1 [239].

Highly elevated expression of certain stress-responsive genes under stress conditions makes their transcripts particularly susceptible to RNA processing defects and, therefore, effective processing mechanisms are necessary to produce functional mature transcripts. Gene encoding HSFA2 has been shown to give rise to different splicing isoforms depending on the environmental temperature. *HsfA2* contains two exons and a single intron. Under moderate heat, an additional exon within the intron is transcribed, introducing a pretermination stop codon (PTC). This *HsfA2-II* variant presents an incomplete DNA binding domain and is degraded through nonsense-mediated decay (NMD) [240]. Severe heat induces the formation of a different splice variant, *HsfA2-III*, that encodes a small, truncated protein due to a cryptic 5${}^{\prime }$ SS in the intron. Interestingly, this isoform can bind the *HsfA2* promoter to activate positive self-regulation.

Sm core protein SmEb, another spliceosome component, is involved in ABA signalling [241]. The expression of SmEb is upregulated after ABA treatment. SmEb enhances the HAB1.1 splicing variant while suppressing HAB1.2 through regulating the alternative splicing of the ABA signalling component HAB1. Contrary to HAB1.2, HAB1.1 overexpression can restore the ABA-hypersensitive phenotype of *smeb* mutants. ABA hypersensitivity of *smeb* mutants is reduced during seed germination when mutations in the transcription factors ABI3, ABI4, or ABI5. SmEb is therefore important for ABA-dependent regulation of seed germination and early seedling growth.

While RNA splicing has been regarded as a posttranscriptional process, recent evidence revealed that the intron could be cotranscriptionally spliced (cotranscriptional splicing, CTS). Cotranscriptional splicing has been reported to be a widespread phenomenon occurring at a high frequency in human cells [242]. It was recently reported that splicing is initiated during transcription for nearly all the introns in *Arabidopsis* [243, 244]. In addition, the processing of alternatively spliced introns was less efficient than constitutively spliced introns. Also, the cotranscriptional splicing was more efficient for protein-coding genes than for those in ncRNAs [243]. In *Arabidopsis*, native elongating transcript sequencing (NET-seq) revealed that phosphorylation of Polymerase II facilitates interaction with the spliceosome, influencing both constitutive and alternative splicing [245, 246]. Additional proteins involved in CTS include the RNA binding protein, HIGH OSMOTIC STRESS GENE EXPRESSION 5 (HOS5) and RS40 and RS41 (two arginine-rich splicing factors), which appear to promote efficient splicing of stress-related genes [247]. CTS efficiency is influenced by the expression level and the number of introns and exons within genes and chromatin modifications [248]. In accordance, a mutant in maize chromatin remodelling complex component ZmCHB101 showed defects in alternative splicing profiles under control and abiotic stress conditions [249]. Altogether, CTS is emerging as an important layer of regulation of alternative splicing, and its impact on abiotic stress responses is under investigation.

Additionally, abiotic stress also triggers alternative polyadenylation. In response to abiotic stress in sorghum, changes in polyadenylation result in the accumulation of nonfunctional transcripts and translational products [250]. Salt stress causes *Arabidopsis* to utilise alternate poly(A) sites in the coding and 5${}^{\prime }$ untranslated regions of transcripts enriched for ABA signalling activities [251]. Plant heat tolerance is likewise adversely regulated by alternative polyadenylation in two rice landraces, Azucena and Tadukan98 [252].

### 5.3. Translational Regulation

Modulation of mRNA translation rates seems to be a conserved feature of cellular responses to diverse stress conditions [253]. The translation is one of the most energy-intensive processes making it the key cellular process to be downregulated under stress conditions. The immediate cellular stress responses occur at the translational apparatus, including ribosomal stalling, translation initiation blocking, and other ribosomal changes. Few reports have elucidated somewhat discordant protein and mRNA expression dependent on the duration, intensity, and type of abiotic stress [254–256]. Translational levels of downstream mORFs are affected by their sequence characteristics such as length, GC content, and minimum free energy that determines the structural stability of RNA secondary structures [254].

In *Arabidopsis*, exposure to heat stress shows similarity with an identified pattern in mammalian cells; induction of 5${}^{\prime }$ ribosome pausing (ribosomal stalling) leads to degradation of mRNA preferentially targeting mRNA encoding HSP70/HSC [257]. This mRNA degradation likely contributes to plant acclimation and survival under chronic heat stress conditions due to XRN4 dysfunction, an exoribonuclease that degrades the mRNA downregulates tolerance of *Arabidopsis* plants to prolonged moderate-high temperature (35°C) exposure [258]. Conversely, the same exoribonuclease degrades mRNA encoding the key heat stress transcription factor, HSFA2, and without functional AtXRN4 gene, plants displayed enhanced survivability following short-term extreme heat stress (43.5°C) exposure [259], pointing to negative impact in plant response to acute heat stress caused by the heat-triggered mRNA. Furthermore, heat stress induces a block in translation initiation leading to preferential storage of mRNA encoding ribosomal protein (RPs) stress granules. These stored mRNAs are released during stress recovery, and their translation is restored by a process dependent on HSP101/CLB1 [260].

### 5.4. Posttranslational Regulation

Protein posttranslational modifications (PTMs), the covalent postsynthetic modifications influence the protein activities, cellular localization, and/or accumulation, thereby playing important functions in stress response regulation [261]. Various abiotic stress conditions are known to induce posttranslational modifications [262]. However, the functional significance of these modifications has not been addressed.

Rapid changes in plant growth behaviour in response to stress conditions are underpinned by the degradation of preexisting regulatory proteins and the synthesis of new ones. The Ubiquitin-Proteasome Pathway (UPP) plays a significant role in this function—allowing rapid response and adaptation of plants to ever-changing environmental cues. The proteolytic function of the UPP involves two discrete stages: ubiquitylation of the substrates and degradation of the tagged protein [263]. E3 Ubiquitin ligases catalyse the attachment of small protein modifier Ubiquitin to target selected proteins for degradation [264]. In consistency with the role of the UPP in plants stress response, a large group of E3 ligases are encoded in plant genomes. The specificity of the ubiquitin-proteasome degradation pathway can be attributed to at least the following proteins—E3 ubiquitin ligase and the matching substrate [265–267]. The stress-related proteins that are potential substrates of ubiquitylation include important TFs, epigenetic regulators, and enzymes involved in ABA signalling and metabolism.

Plant response to environmental stresses can be expedited by conjugating of Small Ubiquitin-like Modifiers (SUMO) to intracellular proteins. SUMO targets are the second most common kind of protein subjected to posttranslational changes. The SUMOylation of protein substrates is significantly enhanced by plant exposure to heat, cold, drought, and oxidative stresses. Short periods of exposure to abiotic stress conditions such as cold, heat, or oxidative stress (H_2_O_2_) trigger the sumoylation of a wide range of substrates [268–272]. Plant recovery from stress conditions is accompanied by rapid desumoylation of this massive pool of sumoylated proteins. SUMOylation is identified as the most significant posttranslational modification during abiotic stresses exposure in crops such as rice [273, 274], tomato [275], maize [276], and soybean [277]. For instance, in cotton, the Rice SUMO E3 LIGASE, OsSIZ1 overexpression enhanced water-deficit tolerance, improved net photosynthetic rate, as well as improved cotton growth and fibre yield [273].

Myristolyation is a protein-lipid modification that plays an essential role in membrane targeting [278]. The ubiquitous eukaryotic enzyme, N-myristoyltransferase, catalyses the myristoylation process. The N-myristoylation is the normal state of *Arabidopsis* phosphatase EGR2 that enables efficient interaction with and inhibition of SnRK2.6 protein kinase [279]. However, cold stress conditions lead to enhancement of EGR2 (Plasma membrane-localized clade-E growth-regulating 2) expression and weakening its interaction with the N-myristoyltransferase NMT1, resulting in the suppression of N-myristoylation of EGR2 [279]. Consequently, EGR2-mediated inhibition of SnRK2.6 activity is released, resulting its regulatory role in freezing tolerance. PTMs also influence the activity of several other proteins that are critical for stress tolerance but are not part of stress signalling. Osmotic stress conditions and ABA-dependent signalling activates SnRK2s protein kinases. The activated SnRK2s then phosphorylate TFs, transporters, and many enzymes, including enzymes associated with maintaining ROS homeostasis and biosynthesis of osmoprotectants/osmolytes [280]. For example, the phosphorylation of SLAC1 triggered by ABA results in stomatal closure due to reduced turgor pressure in guard cells [281]. Stress-induced accumulation of ROS, NOx (nitrogen oxides), and SO_2_ (sulphur dioxide) can trigger PTMs involving redox-based modifications such as oxidation, S-nitrosylation, nitration, glycations, S-glutathionylation, persulfidation, and carbonylation [282–284]. Nitric oxide-based modification is an important PTM involving cysteine residue modification of target proteins called S-nitrosylation [285]. In *Arabidopsis*, S-nitrosylation of PROTEIN ARGININE METHYLTRANSFERASE5 (PRMT5) enhances its methyltransferase activity essential for accurate splicing of pre-mRNAs upon stress exposure [286].

### 5.5. Epigenetic Processes in Abiotic Stress Tolerance

Epigenetic modifications lead to changes in specific chromatin domains to permit or repress transcription of a certain set of genes. Recently, it has been reported that a reversible epigenetic regulation of chromatin architecture can underpin genomic, transcriptional, and metabolic changes for different cellular processes [287–289]. Investigations on epigenetic control of abiotic stress response in plants have uncovered an additional layer of control exerted by epigenetic elements [290, 291]. The main epigenetic control elements include histone variants, histone modifications, chromatin remodelling, regulatory RNAs (e.g., noncoding RNA), and DNA methylation [292].

Histone acetylation is modulated by histone acetyltransferases (HATs) and histone deacetylases (HDACs). These two counteracting enzyme families regulate the acetylation state of lysine residues, particularly those within the N-terminal extensions of core histone proteins [293]. In *Arabidopsis*, salinity stress induces expression of histone acetyltransferase *GCN5*, and plants with mutations in this gene show enhanced salt stress sensitivity due to a deformation of cell wall integrity. *GCN5* exerts its control via activation of a *CTL1*, a gene encoding a chitinase-like (CTL) protein through H3K9/K14 acetylation [294]. CTL1 plays a crucial role in cell walls biosynthesis and salt stress tolerance. In addition, *gcn5* mutants exhibit severe heat stress sensitivity [295]. Hu et al. [295] propose that *GCN5* mediates H3K9/K14ac enrichment in *HsfA3* promoter and *ULTRAVIOLET HYPERSENSITIVE6* genes. Transcriptome studies point towards the important role of HATs in the abiotic stress response of crop plants [296].

Histone deacetylases (HDACs) also play a significant role in drought and salt stress responses. The *Arabidopsis* genome contains 18 HDACs, and out of these, HDA9 and HDA19 enhance salt sensitivity [297–299], while HDA6, HD2C, and HD2D enhance salt tolerance [298, 300]. HDA19 modulates ABA signalling by regulating the expression level of ABA receptor genes [297].

Histone methyltransferases mediate the transfer of the methyl group to lysine residues of histones, whereas the removal is mediated by demethylases (HDM) [301, 302]. HDMs are classified into two groups, Lys-specific demethylases (LSD), and JumonjiC (JmjC) domain-containing protein family. JMJ15 demethylases have been reported to enhance salinity tolerance, while JMJ17 demethylases are reported to participate in water-deficit conditions [303, 304].

Histone ubiquitination is a reversible epigenetic modification that adds or removes the ubiquitin moiety from histones [305]. It has been shown that monoubiquitination of H2B is associated with abiotic stress response in rice and *Arabidopsis*. Enhanced drought tolerance has been observed in cotton plants overexpressing an *Arabidopsis* E3 ligase *AtHUB2* [306]. In rice, the *OsHUB2* overexpression unravelled that H2Bub1 (Histone H2B monoubiquitination) plays a role in positively modulating of ABA sensitivity and resistance to drought stress [307].

In plants, abiotic stress can induce the synthesis of histone variants that can modify the chromatin architecture by replacing their canonical forms [308]. Histone variant H2A.Z can exert positive or negative control on transcription depending upon its accumulation in gene bodies on the transcriptional start site [309]. The variant H2A.Z plays a significant role in regulating plant responses to cold and heat stress conditions [310].

Investigations on how histone modifiers are targeted to specific gene loci have revealed that some histone modifiers are targeted to specific chromatin sites via transcription factors. At the same time, in other cases, the targeting is achieved through lncRNAs [311, 312]. In the case of rice, INDETERMINATE SPIKELET1 (IDS1) and in *Arabidopsis* MYB96 are reported to recruit HDAC in response to high-salt and drought conditions, respectively [313, 314]. In the case of poplar (*Populus trichocarpa*), AREB1 acts as a recruiter of HAT in drought stress response [315]. Furthermore, in rice, OsbZIP46 acts as a recruiter of both an H2B ubiquitinase and deubiquitinase in response to water-deficit conditions [307].

DNA methylation, a conserved epigenetic mechanism, has also been reported to regulate abiotic stress response in plants. DNA methylation in plants mainly occurs by adding of a methyl group to the 5^th^ position of the Cytosine’s pyrimidine ring (5mC: 5-methylcytosine) or the 6^th^ position of the Adenine’s purine ring (6mA: N6-methyladenine). In plants, the RNA-directed DNA methylation (RdDM) pathway establishes *de novo* 5mC DNA methylation, and various DNA methyltransferases such as DOMAINS REARRANGED METHYLTRANSFERASE 2 (DRM2) maintain DNA methylation on the sequence contexts CG, CHG (H can be A, C, or T), and CHH [316, 317]. Diverse alteration of 5mC DNA methylation in response to different abiotic stress has been reported in crop species [318]. In response to heat stress, higher DNA methylation levels are reported in the anthers of a heat-tolerant cotton line compared to a heat-sensitive line [319, 320]. Contrary to this, drought-sensitive genotypes exhibit an increase in the DNA methylation levels in rice, whereas drought-tolerant genotypes exhibit hypomethylation [321].

Furthermore, DNA methylation of key abiotic stress regulatory genes is potentially associated with the stress response. For example, salt stress significantly decreases the 5mC levels at the promoter of TF GmMYB84 in soybean, which potentially upregulates its expression. GmMYB84 interacts with the cis-regulatory regions of K^+^ TRANSPORTER 1 (GmAKT1), thereby enhancing salt stress tolerance [322]. Similarly, in *Arabidopsis*, variation in ICE1 5mC methylation most likely contributes to phenotypic variability in freezing tolerance [323]. Compared to the 5mC DNA methylation, the regulation of abiotic stress by 6mA DNA methylation is reported by very few studies. In rice, heat and salt stress response is associated with increased 6mA levels, and the fold change is more significant in the tolerant cultivars [324]. It is, however, unknown whether heat or salt stress-induced 6mA upregulation is preserved across species.

Recent studies have addressed the role of selected DNA methylation-related genes in regulating the abiotic stress response. Arabidopsis plants lacking NRPD2, the shared second-largest component of PoI IV and Pol V, are highly susceptible to acute heat stress (42°C for 24–34 h). Additionally, the loss of function of RdDM components RNA-DEPENDENT RNA POLYMERASE 2 (RDR2), DICER-LIKE 3 (DCL3), and ARGONAUTE 4 (AGO4) resulted in a significant reduction in basal thermotolerance [325]. Arabidopsis plants with a mutation in RDM16, which encodes a pre-mRNA splicing factor 3 involved in the RdDM pathway, are hypersensitive to salt stress [326]. Additionally, suppressing *SlAGO4A*, a critical component of the RdDM pathway, significantly increased salt and drought tolerance in tomatoes compared to nontransgenic and *SlAGO4A* overexpressing plants [327]. Further research utilising forward and reverse genetic techniques and genome-wide profiling is required to elucidate the functions of DNA methylation-related genes in abiotic stress response regulation.

## 6. Role of ncRNAs in Abiotic Stress Response

Based on their origin, biogenesis, and mode of action, noncoding RNAs (ncRNAs) have been divided into various groups [328]. The two most common types of ncRNA transcripts are housekeeping and regulatory ncRNAs. MicroRNAs (miRNAs), short interfering RNAs (siRNAs), piwi-interacting RNAs (piRNAs), circular RNAs (circRNAs), and long noncoding RNAs (lncRNAs) are all examples of regulatory ncRNAs. These regulatory ncRNAs are transcribed from DNA but cannot be translated into proteins [329]. Differential expression of ncRNAs in response to unfavourable environmental conditions has been documented in several studies. ncRNAs can regulate gene expression in interconnected cellular networks, or they can respond to abiotic stress directly.

### 6.1. miRNAs

Research during the past decade has progressively emphasised the importance of miRNAs in plants’ responses to abiotic stress as a rapid, effective, and tissue-specific method for restoring normal plant function [330, 331]. While certain miRNAs are reported to be stress-specific, others are differentially expressed under different abiotic stresses. Additionally, certain abiotic stress-responsive miRNAs are evolutionarily conserved across plant species. For instance, in response to drought stress, upregulation of miR160, miR162, miR395, and miR827, whereas downregulation of miR166, miR172, miR397, miR827, and miR1432 in maize, rice, wheat, and *Arabidopsis* has been reported [332–335]. miRNAs have emerged as promising targets for improving plants’ ability to respond to and endure abiotic stress.

miRNAs are suggested to play a key role at the crossroads of complex stress-responsive gene regulatory networks. miRNAs target gene expression via mRNA cleavage, translational repression, and DNA methylation [336]. Thus, if a miRNA is upregulated in response to abiotic stress, it will downregulate the expression of its target genes. In contrast, if a miRNA is downregulated, it will accumulate the target mRNAs. Furthermore, the presence of complex regulatory networks involving stress-responsive TFs and miRNA has been suggested in plants [337]. In rice, the miR164 and NAC TF network are essential for regulating drought tolerance, as highlighted in drought-sensitive transgenic lines where miR164 overexpression displays associated suppression of target NAC TFs [338]. Several other TFs, including ARF, AP2, HD-ZIP III, HSF, TCP/PCF, NF-YA5, WRKY/GRF, MYB, NAC, and SPL, have been associated with miRNA:TF module regulating abiotic stress response [339]. These networks orchestrate abiotic stress signalling by altering various metabolic, signalling, molecular, and regulatory pathways. For example, miR159, miR160, miR164, and miR167 are potentially linked to ABA, GA, JA, SA, auxin, and other key phytohormone signalling pathways [340, 341].

### 6.2. lncRNAs

Plant lncRNAs have recently been identified for their plausible role in regulating of abiotic stress [342]. lncRNAs are not highly conserved, and their expression pattern is species-dependent, as a consequence, identifying conserved lncRNAs among different plant species is less likely. Furthermore, in response to abiotic stress, in contrast to protein-coding genes, lncRNAs display expression patterns highly specific to tissue and stage of development [343, 344]. This disparity associated with the differential number of identified lncRNAs across plants species may potentially be explained by variations in the techniques used to screen and identify lncRNAs. For instance, a report in *Arabidopsis* identified 1832 lncRNAs to be sensitive to drought, cold, salt, and ABA, but the technique only identified intergenic lncRNAs [345]. On the other hand, in *Medicago truncatula* 5634 lncRNAs responsive to drought were identified based on an approach to identify all classes of lncRNAs [346].

Mechanism of abiotic stress response regulation mediated by lncRNAs can be varied [342, 347]. Certain plant lncRNAs engage in the abiotic stress response by mimicking their targets by functioning as miRNA-targeted competitive endogenous RNAs (ceRNAs), preventing miRNA interactions with their targets. For instance, in *B. rapa*, two lncRNAs were identified as endogenous target mimics for miR164a in response to high temperature [348]. Certain lncRNAs use the RdDM (RNA-directed DNA methylation) silencing pathway to react to environmental stress [349]. An Arabidopsis long intergenic noncoding RNA induced by auxin—AUXIN REGULATED PROMOTER LOOP (APOLO)—was transcribed by RNA polymerases II and V [350]. APOLO’s dual transcription controls the formation of a chromatin loop that includes the promoter of its nearby gene PINOID (PID), a major regulator of polar auxin transport, causing its transcripts to be downregulated. APOLO may also target distant nonassociated loci by generating R-loops (DNA-RNA duplexes), or APOLO-mediated LIKE HETEROCHROMATIC PROTEIN 1 (LHP1) decoy may induce target locus transcription initiation, thus, altering local 3D chromatin conformation and coregulation of auxin-responsive genes [351]. Further research is warranted to unravel the apparent complexity of RdDM and its role activating stress-responsive genes.

Furthermore, in cold weather, COLD INDUCED LONG ANTISENSE INTRAGENIC RNAs (COOLAIR) and COLD ASSISTED INTRONIC NON-CODING RNA (COLDAIR) lncRNAs are reported to assist blooming in plants [352, 353]. COOLAIR is an alternatively spliced natural antisense transcript lncRNA transcribed from the *FLC* (a regulator of flowering time) gene, whereas COLDAIR is transcribed from the first intron of the *FLC* gene. FLC encodes a MADS-box TF that represses floral induction [354]. COOLAIR and COLDAIR expression are reported to inhibit FLC expression in cold-stressed *Arabidopsis* through lncRNA-mediated chromatin changes (lncR2Epi). COOLAIR represses the FLC locus during the early stages of cold stress via modifying the FLC locus’ chromatin by decreasing the active histone mark H3K36me3 and increasing the repressive histone mark H3K27me3 during vernalization. COLDAIR represses FLC by engaging the Polycomb Repressive Complex 2 (PRC2), which assists in FLC locus chromatin modification by increasing H3K27me3 methylation. An additional polycomb-binding lncRNA, COLDWRAP, is also suggested to contribute to the stable suppression of FLC during *Arabidopsis*vernalization [355].

DROUGHT INDUCED lncRNA (DRIR) is a lncRNA that responds to high salt and water-deficit stress in *Arabidopsis* [356]. DRIR acts as a positive stress regulator and transcriptionally regulates several drought stress-responsive genes, including but not limited to signalling genes (ABI5, P5CS1, RD29A, and RD29B), aquaporin genes (NIP1, TIP4), annexin gene (ANNAT7), and TFs (NAC3, WARKY8). Further, drought and salinity tolerance was enhanced in DRIR overexpressed plants. Although lncRNAs have been shown to be abiotic stress-responsive, their functional characterisation is mostly missing.

### 6.3. circRNAs

Abiotic stress control by circRNAs in plants has received less attention so far. Stress-responsive circRNA expression in agricultural plants has only been documented in a few studies. circRNAs that respond to drought and heat stress have been detected in *Arabidopsis* and a few economically important crops [357, 358]. Available research indicates that circRNA potentially modulates gene expression by acting as miRNA sponges or regulating translation [359–361]. The exact mechanism of abiotic stress response regulation by circRNA in plants is unknown, and recent research suggests that circRNAs either function as miRNA sponges or limit the synthesis of sRNAs, thus, preserving stress-sensitive transcripts from gene silencing [362, 363]. In *Arabidopsis*, overexpression of circGORK (Guard cell outward-rectifying K^+^-channel) led to the activation of many ABA-sensitive genes in transgenic lines indicating a positive modulation of drought tolerance [358]. Furthermore, the overexpression of Vv-circATS1 sourced from grape resulted in increased cold tolerance in *Arabidopsis*, while its linear equivalent had no effect [364]. These studies offer practical methods and a framework for elucidating the function of circRNAs in stress response control.

## 7. Engineering Abiotic Stress Tolerance in Crops

As discussed in previous sections, a sensing/perception of abiotic stress by plants activating complex interconnected regulatory networks that govern stress-responsive gene expression to counteract the negative consequences of abiotic stress exposure, thereby maintaining cellular equilibrium. There is a crosstalk between various regulatory, metabolic, and developmental processes. As a result, while acting upstream in the signalling network may enhance tolerance to certain types of stress, there is an enhanced risk of causing undesirable pleiotropic consequences such as growth defects and developmental alterations. Targeting the expression of direct-action genes usually only improves performance in response to specific types of stress [365]. These considerations are especially important since plants in natural habitats are frequently exposed to multiple stressors, such as heat and drought, that can have synergistic, neutral, or even antagonistic effects.

Successful transfer of characteristics proved to be effective in the lab; the performance of the improved abiotic stress tolerance trait in the field has proven difficult. With several studies reporting the development of abiotic stress-tolerant plants, most research has focused on vegetative development stages such as leaf or root physiology instead of reproductive stages leading to seed formation, development, and maturation. A stress scenario during the flowering phase leads to hefty yield penalties [366, 367]; therefore, the plant development stage is also a key element. Additionally, genetically engineering plants to impart abiotic stress tolerance entails intervening at many levels of the abiotic stress response (Table 1).

**Table 1:** List of representative stress-tolerant crops developed by genetic modification (GM). OE: overexpression; TSE: tissue-specific expression; COE: co-overexpression.

Crop	GM	Promoter	Gene	Details of the gene	Source	Tolerance trait	Physiological benefit in response to stress	Reference
Barley	OE	35S	*AtAVP1*	H^+^-pyrophosphatase	*Arabidopsis*	Salt	In field, higher grain yield and higher shoot biomass	[431]
Rice	OE	Maize Ubi1	*AtHsp101*	Chaperone	*Arabidopsis*	Heat	Better growth performance in the recovery phase following the stress	[384]
Rice	OE	Maize Ubi1	*TaMBF1c*	MBF TF	Wheat	Heat	Tolerance during both vegetative and reproductive stages	[369]
Rice	OE	Maize Ubi1	*OsRab7*	Small GTP-binding protein	Rice	Heat	Higher yield	[432]
Rice	OE		*SNAC3*	NAC TF	Rice	Heat, drought	Better survival rate	[433]
Rice	COE	Tissue-specific or stress-dependent	*otsA and otsB* (Fusion)	Trehalose biosynthetic genes	*E. coli*	Salt, cold, drought	Sustained plant growth, less photo-oxidative damage, and more favourable mineral balance	[386]
Rice	OE	ABA-inducible promoter	*SAMDC*	S-adenosylmethionine decarboxylase	*Tritordeum*	Salt	Increased seedling growth	[434]
Rice	OE	35S	*P5CS*	Proline synthesis gene	*Vigna aconitifolia*	Salt	Wild type died after 10 days of salt stress whereas, even after 4 weeks of stress transgenics were capable of flowering and seed-setting	[435]
Rice	OE	35S	*PpENA1*	Na^+^ pumping ATPase	*Physcomitrella patens*	Salt	Higher biomass production	[436]
Rice	CE	35S (pyramiding)	*SaSRP3-1, SaVHAc1*	Salt Responsive Protein 3-1, Vacuolar H^+^-ATPase subunit c1	*Spartina alterniflora*	Salt	Higher grain yield weight, and improved shoot and root growth	[437]
Rice	OE	35S	*Gly I, Gly II*	Glyoxalase pathway genes	Brassica, Rice	Multiple stress	High shoot and root biomass	[438]
Rice	OE	Native promoter	*SNAC1*	NAC TF	Rice	Drought	In field 22–34% higher seed setting (stress during reproductive stage)	[368]
Rice	OE	Stress-inducible *rd29A* promoter	*AtDREB1A*	AP2/ERF TF	*Arabidopsis*	Drought	Tolerant during both the vegetative and reproductive stages	[391]
Rice	TSE	Root specific *RCc3* promoter	*OsERF71*	AP2/ERF TF	Rice	Drought	23% to 42% higher grain yield over WT or whole-body OE transgenic lines	[370]
Rice	OE	PGD1	*OsNAC14*	NAC TF	Rice	Drought	In field higher number of panicle and filling rate	[439]
Rice	OE	Rice Actin1P/LEA3-1P (stress inducible)	*NPK1*	MAPKKK	Tobacco	Drought	In field higher seed setting rate	[374]
Rice	OE	Rice Actin1P/LEA3-1P (stress inducible)	*SOS2*	Serine/threonine kinase	*Arabidopsis*	Drought	In field higher seed setting rate	[374]
Rice	OE	rice Actin1P/LEA3-1P (stress inducible)	*AtDREB1A*	AP2/ERF TF	*Arabidopsis*	Drought	In field higher seed setting rate	[374]
Rice	OE	Rice Actin1P/LEA3-1P (stress inducible)	*Zat10*	C2H2-EAR zinc finger	Rice	Drought	In field higher seed setting rate	[374]
Rice	OE	Rice Actin1P/LEA3-1P (stress inducible)	*LOS5*	Molybdenum cofactor sulfurase	*Arabidopsis*	Drought	In field higher seed setting rate	[374]
Rice	OE	Rice Actin1P	*AtNHX1*	Na^+^/H^+^ antiporter	*Arabidopsis*	Drought	In field higher seed setting rate	[374]
Rice	OE	Maize Ubi1	*OsbZIP23*	bZIP TF	Rice	Salt, drought	Better growth	[440]
Rice	RNAi	Maize Ubi1	*SQS*	Squalene synthase	Maize	Drought	14–39% higher yield	[441]
Rice	OE		*OsSKIPa*	Rice homolog of human Ski-interacting protein	Rice	Drought	Resistant at both vegetative and reproductive stages	[442]
Rice	OE	2X35S	*OsLEA3-2*	Late embryogenesis abundant (LEA) proteins	Rice	Salt, drought	Stronger growth performance, recovery after 20 days of drought stress	[443]
Rice	OE	35S, drought inducible promoter	*OsLEA3-1*	Late embryogenesis abundant (LEA) proteins	Rice	Drought	Higher relative yield (yield under drought stress treatment/yield under normal growth conditions)	[383]
Rice	OE	35S	*OsIF*	Intermediate filament	Rice	Heat, salt	Maintenance of yield	[444]
Rice	OE	Maize Ubi1	*OsMYB55*	MYB TF	Rice	Heat	Lesser yield reduction	[445]
Rice	COE	35S	*OsbZIP46CA1, SAPK6*	bZIP TF, ABA-Activated Protein Kinase	Rice	Drought, cold, heat	Higher yield, biomass, spikelet number, and grain number	[446]
Rice	OE	35S	*RGB1*	Beta subunit of G protein	Rice	Heat, salt	Higher germination rate, root length, shoot length and plant height	[447]
Rice	OE	Promoter of *Rca-a* from *Oryza meridionalis*	*Rubisco activase*	Rubisco activase	*Oryza australiensis*	Heat	Higher yield (stress during vegetative stage)	[448]
Rice	COE	Rice Cab promoter	*Rubisco, Rubisco activase*	Rubisco, Rubisco activase	Rice, maize	Heat	Higher biomass and improved photosynthesis	[449]
Rice	OE	Actin	*miRNA319*	miRNA	Rice	Cold	Increased leaf width and vein number, and better acclimation	[378]
Rice	OE	35S	*TERF2*	AP2/ERF TF	Tomato	Cold	Better survival rate	[450]
Rice	OE		*COLD1*	Regulator of G-protein signalling	Rice	Cold	Enhanced chilling tolerance	[451]
Rice	OE	Ubiquitin	*MYBS3*	MYB TF	Rice	Cold	In field no yield penalty	[452]
Rice	OE	Ubiquitin	*OsMYB3R-2*	MYB TF	Rice	Cold	Higher cold resistance, higher levels of proline	[453]
Rice & Tomato (small fruit species)	OE	35S	*ERECTA*	LRR-RLK	*Arabidopsis*	Heat	Increased biomass and yield	[454]
Rice	OE		*OsMADS57*	MADS-box TF	Rice	Cold	Maintenance rice tiller growth	[455]
Wheat	OE	Ubiquitin	*betA*	Choline dehydrogenase	*E. coli*	Salt	In field higher grain yields, more tillers, and higher germination rates	[456]
Wheat	OE	pIND	*HaHB4* (mutated)	Homeodomain-leucine zipper I family gene	*Helianthus annus*	Drought	In field higher spikelet numbers per spike, tillers per plant, and fertile florets per plant	[457]
Wheat	OE	Barley *Dhn8s*	*TaHsfC2a*	HSF TF	Wheat	Heat	Enhanced thermotolerance	[458]
Wheat	OE	Maize Ubi1	*TaFER-5B*	Ferritin gene	Wheat	Heat	Enhanced stress tolerance	[459]
Wheat	OE	Hordein B1 promoter	*HvSUT1*	Sucrose Transporter	Barley	Heat	Better performance for many yield-related traits and enhanced sucrose transport	[460]
Wheat	OE	Maize Ubi1	*EF-Tu*	Elongation factor	Maize	Heat	Reduced heat injury to photosynthetic membranes (thylakoids), and enhanced rate of CO_2_ fixation	[461]
Wheat	OE	Drought inducible Barley HVA1s	*Hsf6a*	HSF TF	Wheat	Heat	Enhanced thermotolerance	[462]
Wheat	OE	35S	*AtWRKY30*	WRKY TF	*Arabidopsis*	Heat, drought	Improved biomass and photosynthesis	[463]
Maize	OE	*Rd29A*	*ZmbZIP4*	bZIP TF	Maize	Salt, drought	Enhanced root development and ABA synthesis	[464]
Maize	CRISPR	Maize *GOS2*	*ARGOS8*	Negative regulator of ethylene signalling	Maize	Drought	In field increased grain yield by five bushels per acre (stress exposure during flowering)	[465]
Maize	OE	35S	*NPK1*	MAPKKK	Tobacco	Drought	No yield loss under stress	[375]
Maize	OE	Novel promoter	*LOS5*	Molybdenum cofactor sulfurase	*Arabidopsis*	Drought	Increased root system development and biomass yield after re-watering	[466]
Maize	OE	Maize Ubi1	*OsMYB55*	MYB TF	Rice	Heat, drought	Higher plant biomass and reduced leaf damage	[467]
Cotton	OE		*AhCMO*	Choline monooxygenase	*Atriplex hortensis*	Salt	Higher seed yield	[385]
Cotton	COE		*AtNHX1, AtAVP1*	Na^+^/H^+^ antiporter, H^+^-pyrophosphatase	*Arabidopsis*	Salt, drought	24% and 35% higher fibre yield under low-irrigation and dryland conditions, respectively	[468]
Cotton	OE	35S	*AtAVP1*	H^+^-pyrophosphatase	*Arabidopsis*	Salt, drought	In field 20% higher yield under dry-land conditions	[469]
Cotton	OE	35S	*GhDof1*	DOF TF	Cotton	Salt, cold	Higher oil content and reduced protein in seeds	[470]
Tomato	CE	35S	*PgNHX1, AVP1*	Na^+^/H^+^ antiporter, H^+^-pyrophosphatase	*Pennisetum glaucum*, *Arabidopsis*	Salt	Better survival rate	[471]
Tomato	OE	35S	*SbNHXLP*	Na^+^/H^+^ antiporter-like protein	*Sorghum bicolor*	Salt	Higher fruit yield	[381]
Tomato	OE		*APX (cytosolic)*	Ascorbate peroxidase	Pea	Heat	Enhanced tolerance to heat stress in lab and field	[382]
Tomato	OE	35S	*SAMDC*	S-adenosyl-l-methionine decarboxylase	*Saccharomyces cerevisiae*	Heat	Improved the efficiency of CO_2_ assimilation	[472]
Tomato	OE	35S	*ShDHN*	Dehydrin	Wild tomato	Cold, drought	Better survival rate	[473]
Tomato	OE	Stress inducible *AtRd29A*	*BoCRP1*	Novel cold-responsive protein1	*Brassica oleracea*	Cold	Higher seed germination, increased root length, reduced membrane damage and increased accumulation of osmo-protectants	[474]
Tomato	OE	35S	*LeCOR413PM2*	Cold-regulated gene	Tomato	Cold	Reduced damage to cell membrane, accumulation of ROS, and photoinhibition of PSII, but also maintain high activity of antioxidant enzymes and content of osmotic regulators	[475]

### 7.1. Regulatory Genes as Potential Bioparts for Imparting Abiotic Stress Tolerance

Modifying the expressions of regulatory genes, such as protein kinases, phosphatase, and transcription factors (TFs), is an efficient strategy to improve stress resistance in plants due to activating stress signals and coordinately regulating many downstream genes. Several TF families, bHLH, MYB, AP2/ERF, bZIP, DREB, NAC, and WRKY, operate as downstream integrators of regulatory networks, influencing the expression of stress-responsive genes in a combinatorial and amplificatory approach. Overexpression of *SNAC1* (a NAC TF) in rice enhanced salt and drought resistance during the vegetative stage and significantly increased yield by 22-34% upon exposure to water-deficit conditions in the field during the reproductive stage [368]. Transgenic rice overexpressing *MBF1c* TF (isolated from wheat) imparted thermotolerance during vegetative and reproductive stages [369]. Furthermore, overexpression of an AP2/ERF TF—*OsERF71*—in rice under the control of a root-specific promoter conferred drought tolerance and enhanced yield by 23-42% upon drought exposure during the reproductive stage [370]. Selected studies utilising TFs for improving abiotic stress tolerance in crops are summarized in Table 1.

Protein kinases and phosphatases play critical roles in the adaptability and growth of plants [371]. Members of the protein kinase families associated with Ca^2+^ mediated signalling are potential candidates for imparting abiotic stress tolerance; for example, overexpression of *OsCIPK03*, *OsCIPK12*, and *OsCIPK15* in rice demonstrated significantly increased cold, drought, and salt stress tolerance, respectively [372]. Protein kinases associated with MAPK cascades can be potentially targeted to enhance tolerance to abiotic stress in plants. *OsMAPK5* overexpressing rice lines exhibited improved drought, salt, and cold stress tolerance [373]. Similarly, Xiao et al. [374] reported higher yield in transgenic rice overexpressing *OsMAPK5* (*NPK1*) upon drought exposure in field conditions. In maize, overexpression of a tobacco *MAPKKK* (*NPK1*) enhanced drought resistance by potentially improving photosynthesis rates [375]. Among protein phosphatases gene candidates, overexpression of *OsPP1a* in rice increased resistance to high salinity by potentially upregulating the expression of *SnRK1A* as well as *OsNAC5* and *OsNAC6* in transgenic lines [376].

Another significant target class in this area is miRNAs, which regulate abiotic stress response by specifically targeting the expression of stress-responsive genes. In rice, salt and alkali responsive osa-MIR393 potentially target expression of stress-responsive genes, namely, phytosulfokine receptor precursor (LOC Os02g06260), putative transport inhibitor response protein (LOC Os05g41010), and oxidoreductase (LOC Os05g05800). Overexpression of osa-MIR393 in rice and Arabidopsis increased the salt and alkali sensitivity, thus, targeting the expression of osa-MIR393 might enhance stress tolerance [377]. Overexpression of osa-MIR319 in rice enhanced cold tolerance after acclimation of rice seedlings to suboptimal temperature [378]. Similarly, overexpression of osa-MIR319 in creeping bentgrass improved drought stress tolerance [379]. Furthermore, miR166 knockdown lines in rice (generated by using the Short Tandem Target Mimic system) exhibited a higher survival rate in response to drought stress. Additionally, these lines showed significantly higher spikelet fertility under drought exposure in field conditions [380]. Thus, by identifying and targeting specific miRNAs implicated in abiotic stress regulation using precise genome editing methods, abiotic stress tolerance in crops might be improved. In addition to the abovementioned bioparts, manipulation of bioparts associated with processes such as PTMs of signalling and regulatory elements, epigenetic modification, among others, provide promising ways to achieve generalised stress tolerance while maintaining a higher control over stress response.

### 7.2. Structural or Functional Genes as Potential Bioparts for Imparting Abiotic Stress Tolerance

Numerous approaches for enhancing abiotic stress resistance in crops have been explored, including overproduction of ion transporters, antioxidant enzymes, chaperones, protective proteins, and enzymes involved in metabolite synthesis. Abiotic stress tolerance can be enhanced or imparted in crops by targeting bioparts that restore cellular ionic and redox homeostasis. Under salt stress, ion transporters have been shown to help preserve cellular ion homoeostasis. For example, transgenic tomatoes overexpressing a Na^+^/H^+^ antiporter-like protein (NHXLP) isolated from *Sorghum bicolor* L. (SbNHXLP) displayed enhanced salt tolerance, decreased Na^+^, and increased K^+^ accumulation in root and floral tissues, indicating its involvement in maintaining ion homoeostasis [381]. Maintaining ROS homeostasis in plant cells by targeting the expression of enzymes in the antioxidant machinery is a prevalent approach for boosting plants’ tolerance to direct and indirect oxidative stress and thus improving plant performance under stress conditions. Increased thermotolerance to heat (40°C) was observed in transgenic tomato overexpressing cytosolic APX, most likely owing to the elimination of excessive damaging ROS (especially H_2_O_2_) [382].

LATE EMBRYO ABUNDANT (LEA) proteins and HSPs (also other chaperones) are produced in response to diverse abiotic stresses and are involved in protecting functional proteins. Overexpression of the *OsLEA3-1* gene in rice significantly increased rice grain yields under water-deficit stress in field conditions [383]. Similarly, *AtHSP101* overexpression in the rice cultivar Pusa basmati 1 increased thermotolerance, and transgenic lines had considerably improved growth performance during the recovery phase following stress [384].

Metabolic engineering of compatible solute accumulation is a widely adopted means of improving crop abiotic stress tolerance. Transgenic cotton lines overexpressing a choline monooxygenase from *Atriplex hortensis* (*AhCMO*) demonstrated significantly higher seed yield under salt stress and were more salt-resistant than wild-type cotton due to increased accumulation of glycine betaine, which protected the cell membrane and photosynthetic ability [385]. Accumulation of another compatible solute—Trehalose—in transgenic rice overexpressing a fusion gene made up of trehalose biosynthetic genes (*otsA* and *otsB*; sourced from *E.coli*) enhanced tolerance to multiple abiotic stress, and transgenic lines displayed sustained plant growth, less photo-oxidative damage, and more favourable mineral balance upon abiotic stress exposure [386].

Studies utilising similar approaches for generating transgenic lines displaying higher survival rates, higher yield, and improved abiotic stress tolerance during both vegetative and reproductive stages in economically important crops are summarized in Table 1.

### 7.3. cis-Regulatory Elements (Promoters) as Potential Bioparts for Imparting Abiotic Stress Tolerance

*cis*-Regulatory sequences are critical for gene regulation because they promote TF recruitment. These sequences may be a potential target for generating nucleotide-level alterations that can potentially increase crop tolerance to abiotic stress. For instance, in *Arabidopsis*, ANAC069 is reported to suppress the expression of various stress-responsive genes such as ROS-scavenging genes, thereby adversely regulating stress response mainly by interacting with C[A/G]CG[T/G] cis-elements [387]. A mutation in this core region might result in the inability of ANAC069 to regulate genes, thus, improving stress tolerance.

Furthermore, *cis*-regulatory sequences are predominantly found in the promoter region of genes. Their presence/absence/variation in position/sequence can affect the expression of the gene, resulting in induction, decrease, or even lack of expression. The most often utilised constitutively overexpressed promoters include the 35S promoter of the cauliflower mosaic virus (CaMV), and promoters are derived from plant actin and ubiquitin genes. The constitutive expression can have unforeseen effects on plant growth and development. It might result in overexpression of a specific transgene at the incorrect developmental stage or in tissues that are not ordinarily expressed. For example, constitutive overexpression of rice DREB1 (*OsDREB1*) in *Arabidopsis* exhibited resistance to salt, cold, and drought [388, 389]; however, these transgenic plants exhibited growth retardations. To address the issues related to constitutive overexpression, stress-inducible or tissue-specific promoters with low background expression or tissue-specific expression under normal growth conditions have been utilised. Stress-induced overexpression of the transcription factor *AtDREB1A* under the control of an Arabidopsis stress-inducible promoter, *AtRD29A*, resulted in increased abiotic stress tolerance in transgenic Arabidopsis and rice, addressing the problem of growth retardation [390, 391].

## 8. Synthetic Biology for Improving or Redesigning Abiotic Stress Tolerance in Plants

Synthetic biology uses fundamental engineering principles by employing naturally existing components for the rational design of new biological modules [392, 393]. Such an approach enables the *de novo* fabrication of new gene circuits and switches and restructuring signalling pathways [394]. This new discipline has already been successful in biotechnological manipulations of bacterial, yeast, and mammalian cell systems to develop new materials, production of chemicals via metabolic engineering, and the design of advanced molecular biology and medicinal applications [395–397]. Synthetic engineering of prokaryotic systems has dominated the field, which may be due to their simple cell structures and well-defined components, rendering them attractive systems for the operation of synthetic circuits. Designing synthetic circuits for eukaryotic systems has proven problematic because of their overwhelming complexity compared to prokaryotes. This issue is particularly relevant to the plant systems where despite rapid DNA assembly throughput, the poor transformation efficiency remains a bottleneck for further advances in synthetic biology [398, 399].

Recent advances in understanding systems biology of plant and application of bioinformatics tools have revealed a thorough understanding of regulatory components and processes operating at the cellular level. Such knowledge empowers the construction of synthetic modules using an infinite source of biological parts. Synthetic biology uses a top-down strategy to change what is already in nature. Synthetic biology diverges from the past biological approaches as it aims to build and develop something novel but still inspired by nature [400]. This is a reductionist approach in which biological systems are broken into building components from which new organisms may be constructed and produced. These biological parts (bioparts) may be put together to produce fundamental biological “devices,” which are the simplest assemblies capable of performing a specific function, such as a simple biological circuit (e.g., an on/off switch) or controlling the translation of a certain protein-coding sequence. These replaceable modules may then be integrated into “systems” within a cell or organism to execute a controlled (programmable) higher-level function, such as producing a metabolite in response to particular environmental inputs [401, 402].

### 8.1. Examples of Plant Biodesign Using Multiple Bioparts

The first application of the synthetic biology approach in plant systems includes the inventing of synthetic regulatory elements (synthetic promoters and cis-elements, synthetic short RNAs) and switches to modify of spatiotemporal gene expression and the engineering of signalling networks [403, 404]. The most common switch used in synthetic plant systems is “based on a mammalian steroid signalling pathway” from rats. The chemical-inducible promoter comprises a dexamethasone-inducible pOp/LhGR switch [405]. In the absence of the inducer, there is a low-level expression of the gene of interest, but in the presence of a steroid called dexamethasone (DEX), a high level of gene expression is induced. The DEX-inducible construct was designed by fusing the steroid-binding domain of the DEX-binding region of the glucocorticoid receptor (GR) of rat with the DNA binding domain of *E. coli lac* repressor and the activation domain of Gal4. A tissue-specific promoter is used to drive the expression of the GR-LhG4 transcription factor that remains in the inactive state in the cytoplasm. The presence of DEX allows fusion protein to move into the nucleus, leading to transcription activation of the reporter gene controlled by a synthetic promoter pOp6 that includes 35S core promoter and six copies of the LhG4 binding site. This approach led to the generation of a suite of transgenic driver lines. Following crossing, these lines can induce tissue-specific expression of the reporter gene in the resulting progeny.

Synthetic biologists have also been working to enhance the photosynthetic rate in plants for maximizing plant productivity [406]. Reengineering the primary photosynthetic enzyme, RuBisCO, is one of the approaches being used for this aim. Because RuBisCO is a key enzyme in all carbon-assimilating activities, an increase in RuBisCO activity will directly bear on plant productivity. There has been a successful report of replacing native RuBisCO in the chloroplasts of tobacco plants chloroplast with an alternative form derived from a cyanobacterial source [407]. Compared to the natural tobacco plant, the transformed one containing cyanobacterial RuBisCO had a higher carbon-assimilating efficiency. It has been shown that increased CO_2_ concentrations in the proximity of the RuBisCO enzyme enhances the efficiency of CO_2_ fixation and thus have a positive implication for plant productivity. The cyanobacteria and algae possess innovative carbon-concentrating mechanisms (CCMs) to enhance RuBisCO efficiency of CO_2_ fixation. Therefore, introducing these microautotrophic CCMs to enhance the photosynthetic ability of plants has shown to be an effective strategy [408, 409]. Plant species with C4 type of photosynthesis have evolved innovative CCM that depends on unique tissue anatomy and metabolic pathways. Engineering C4 type of CCMs into less efficient C3 plants via synthetic biology has been the key goal [410]. However, changing C4 photosynthesis into C3 plants remains challenging as much remains to be discovered regarding genes and gene functions underlying the C4 pathway.

Photorespiration is another natural process where RuBisCO binds oxygen resulting in the release of CO_2_ from plants. To address this issue, enzymes that can convert glycolate to glycol-aldehyde have been engineered using synthetic biology approach. Under *in vitro* conditions, these engineered enzymes and other endogenous enzymes could recycle glycolate straight to RuBP (Ribulose 1,5-bisphosphate) without releasing CO_2_ [411]. *In vivo*, several studies have shown that tailored carbon-conserving Calvin cycles may synthesise acetyl-CoA directly from C3 sugars without releasing CO_2_ [412]. Another successful example has been the reconstruction of the CETCH cycle, a synthetic pathway for carbon-fixation through a highly efficient reductive carboxylation process. A synthetic carbon-fixing pathway was engineered optimizing 17 enzymes from nine different organisms [413]. Hence, the above approaches have shown to be highly valuable in enhancing plant yield by reengineering the critical process of carbon fixation in plants.

The creation of plant sentinel biosensors is also a key growing application of synthetic biology in the plant area [12]. The design of the plant biosensors is based on cellular physiological mechanisms that occur naturally in plants or have been artificially developed. They offer various advantages, including better stability and enzyme activity, and being less expensive and time-consuming, making them ideal for application. The development of these whole-plant biosensors relies on a genetic circuit comprising genetically encoded components, which include promoters that respond to external inputs. The invention of such a biosensor for detecting 2,4,6-trinitrotoluene (TNT) explosive is one such successful example of a plant biosensor [414]. This approach was developed a TNT receptor from bacteria into the plant’s degreening gene circuit, triggering the activation of rapid chlorophyll breakdown whenever TNT is detected, resulting in a visible colour shift for simple identification.

Similarly, crops with efficient water utilisation capacity are being developed by controlling the production of ABA via an engineered PRY1 receptor with sensitivity to a fungicide Mandipropomid. The spray of this agrochemical on the plants under drought conditions activates ABA [415]. These modified plants require less water and are more resistant to stress conditions. Synthetic biologists have built a number of very sensitive and durable plant sensors that can track the system's transcriptional output. The stimulation of this mechanism by cytokinin signalling is one example [416]. Cytokinins are a class of plant hormones, playing an essential role in plant physiology and growth; thus, such monitoring sensors might improve understanding of the complex plant developmental mechanisms. Another essential plant hormone, auxin, has also been effectively monitored using synthetic circuits [417]. It has become simpler for plant physiologists to comprehend complicated physiological processes by offering help to construct and regulate responses by critical elements of a plant system, thanks to these sophisticated genetic circuits. Thus, the development of biosensors for the examination of molecular and physical cue perception and signalling relays will aid in the knowledge of stress regulation networks and, as a result, will make it easier to identify effective intervention areas for genetic engineering procedures.

In addition to providing proof of concept, the development of these synthetic techniques offers increasing prospects to reprogram plant development and metabolism and improve agricultural traits. Because these synthetic devices and platforms are plug-and-play, they have the potential to modulate gene expression patterns at numerous levels while not interfering with plant growth, development, or fitness [18, 403]. Even though most plant synthetic biology tools have been designed in well-studied model plants, *Arabidopsis*, and tobacco, and such engineered devices are being progressively adapted for crops.

### 8.2. Perspectives on Improving or Redesigning Abiotic Stress Tolerance through Biodesign

Combining synthetic biology approaches with existing genetic engineering practices offers enticing opportunities for the rational development of abiotic stress-resistant crops. Orthogonality, or the capacity of genetic components and circuits to operating independently of one another and the host’s regulatory activities, is crucial in synthetic biology [418]. Orthogonal components, most commonly bacterial, yeast, or plant viral sequences, can be borrowed in whole or in part from systems other than the intended host species. In this direction, many useful plant bioparts have been sourced from bacterial and viral plant pathogens [18]. Algal, fungal, or photosynthetic bacteria can offer regulatory components that confer sensitivity to stimuli that plants regularly encounter, such as light, drought, and temperature, resulting in plant-like responses in an orthogonal manner.

Synthetic promoters and TFs must be well designed and employed to control of gene expression using the endogenous plant cell transcriptional machinery [419, 420]. Synthetic promoter design entails inserting noncoding cis-regulatory areas (promoter motifs) into existing promoters, either alone or in combination [421, 422]. Promoter motifs are sourced from their native promoters. They are placed upstream of a core promoter that often comprises a TATA box to constitute a transcription preinitiation complex with RNA polymerase II and generic TFs. The minimal 35S promoter has been employed widely; however, several other minimal plant promoters have also been functionally validated. Synthetic promoters are designed and have been tested in a variety of plant species; the design includes synthetic constitutive, tissue-specific, bidirectional, biotic-, abiotic-, or chemical-inducible/responsive promoters [419, 423]. Such synthetic promoters can be used as critical components of genetic circuits for abiotic stress tolerance machinery regulation.

Transcription factors (TFs) play an essential role as regulators of stress-related gene expression (Section 5.1). Each TF has a DNA binding domain and an activation domain that engages the cell’s transcription machinery, reflecting a unique modular architecture. These domains can be constructed in a plug-and-play manner to generate synthetic TFs. By integrating distinct activation, repression, and DNA-binding domains from yeast, a library of synthetic transcription regulators was recently produced, which was subsequently employed for transient gene expression in tobacco leaves and the generation of stably transformed *Arabidopsis* plants [424]. A systematic technique of screening synthetic regulators increased the number of DNA parts tested significantly, and it was easily adaptable to evaluate diverse transcription regulators in different plant species. Synthetic TFs can also be utilised to regulate the expression of numerous genes in tissue-specific and environmentally sensitive ways [420]. Thus, synthetic TFs can be designed to target broad-spectrum stress-responsive genes and therefore can act as potential regulators of genetic circuits whose function can be switched on/off in response to environmental cues.

Synthetic transcription regulators can also be constructed to use epigenetics to control gene expression at the transcriptional level. In transgenic *Arabidopsis* overexpressing these synthetic regulators, Lee et al.[425] reported novel CRISPR-based toolbox for targeted controlling gene expression at transcriptional and epigenetic levels. The authors coupled dCas9, a variant of Cas9 protein lacking nuclease activity, with several regulatory domains for epigenetic regulation of endogenous FLOWERING LOCUS T (FT, master regulator of flowering) expression. Variations in FT expression and/or epigenetic state given by synthetic regulators were linked to changes in flowering time in transgenic *Arabidopsis* lines. Similarly, after further refinement, this strategy could be utilised to maximize the epigenetic control of stress-responsive genes or master stress regulatory genes that are either less epigenetically regulated or regulated by several epigenetic regulators.

Another recently pursued technique is applying riboswitch, a stem-loop RNA structure with regulatory and ligand-binding domains, to modulate mRNA stability and translation in plants [426]. A synthetic riboswitch library with theophylline acting as a ligand has been created for *Arabidopsis* to regulate endogenous and transgenes via posttranscriptional regulation [427]. These riboswitches regulate mRNA stability reliably and efficiently. There is potential to develop novel riboswitches to control gene expression provided their ligands have no influence on plant growth and development and/or are benign for the environment, including humans. This method might potentially be used to create bioparts that can control abiotic stress responses in a spatiotemporal manner.

Engineering the crassulacean acid metabolism (CAM) pathway in plants to improve water-use efficiency and thrive in water-scarce conditions like semiarid deserts is another potential approach for imparting stress tolerance [428]. Drought-tolerance methods observed in resurrection plants, known to tolerate severe drought, or evolutionarily distant creatures with a capacity to anhydrobiosis and survive extreme desiccation, might be engineered using more advanced synthetic biology approaches [429]. Similarly, a sophisticated method to develop a dynamic multilayer protective response regulated, maybe through the circadian clock [430], might permit optimal energy usage by synchronising the abiotic stress-protective response with the diurnal cycle.

## 9. Conclusions

A deep understanding of plant abiotic stress perception, signalling, and response processes is a prerequisite for crops that can maintain yield stability under stress conditions. In recent years, intensive “omics” based investigations have revealed activation of complex stress-responsive regulatory networks upon the perception of external stressors by plants. These interconnected networks involve various biological parts such as sensor proteins, enzymes, transcription factors, epigenetic, and posttranslational modifiers. While genetic engineering approaches involving the modulation of individual bio parts have yielded promising outcomes, the rational design of stress-responsive genetic circuits based on synthetic biology principles is urgently required.

Various genes that can be selected as bioparts for the assembly of stress-protective genetic circuits have been identified. Genes that play significant roles in plant abiotic stress tolerance as functionally tested in transgenic plants are summarized in table, providing a valuable database of bioparts for the rational design of synthetic circuits. These include genes encoding transcription factors, chaperones, stress sensor protein kinases, enzymes that can scavenge reactive oxygen species, and enzymes that promote the accumulation of protective osmolytes.

Besides protein-coding genes, several noncoding RNAs have emerged as potential bioparts to be deployed as tools for enhancing plant abiotic stress tolerance. Among noncoding RNAs, various miRNAs have been functionally validated for imparting abiotic stress tolerance. One of the main issues of concern is that while enhancing the ability of plants to tolerate stress conditions, the constitutive expression of protective genes can have detrimental effects on plant growth phenotype and yield. An on-demand protective functional module that gets activated in a spatially and temporally regulated manner can be fabricated using conditional or tissue-specific promoters is desirable to avoid undesirable consequences. Also, as mentioned, the use of transcription factors or other regulatory components upstream of the protective network enhances the risk of unwanted pleiotropic effects. Additional research is thus warranted to characterise the function of additional direct action candidate genes based on omics and comparative genomics approaches.

Another consideration for designing abiotic stress-tolerant crop plants is the combinatorial action of various stressors in field situations. As discussed, these combined stresses such as heat and drought can have synergistic negative consequences for plant growth and yield. Further, there is a gap in our knowledge regarding stress-responsive regulatory circuits required to protect reproductive development in plants. It has become clear that exposure to environmental stressors such as heat and drought during the flowering phase of plant growth can lead to male sterility, failure of fertilization, and seed set. Thus, a priority is to design protective gene regulatory circuits that are temporally activated in the target reproductive tissues in responding to abiotic stresses. Thus, one of the primary foci for future research in abiotic stress tolerance is to achieve a comprehensive picture of the stress vulnerability of plant reproductive cells. The use of single-cell transcriptomics will enable uncovering of potentially significant cell-specific stress-responsive genes that may be yet undiscovered.

Translation of synthetic biology approaches in plant systems depends upon efficient protocols for genetic transformation and control over the expression of inserted genes. Fortunately, for most of the major food crops such as wheat, corn, canola, soybean, and relatively facile methods of transgene addition are now available. The CRISPR technology for genome editing has already been implemented in major crop genera. The availability of diverse methodologies for genome manipulation and the engineering of synthetic circuits hold the potential for ushering in a new era for developing crop genotypes that can sustain yield stability in the face of multiple abiotic stress linked with climate challenges to agricultural productivity.

## Data Availability

Not applicable.
